# A Molecular Nanotube with Three-Dimensional π-Conjugation**

**DOI:** 10.1002/anie.201502735

**Published:** 2015-05-07

**Authors:** Patrik Neuhaus, Arjen Cnossen, Juliane Q Gong, Laura M Herz, Harry L Anderson

**Affiliations:** Department of Chemistry, University of Oxford, Chemistry Research LaboratoryOxford, OX1 3TA (UK); Department of Physics, University of Oxford, Clarendon LaboratoryParks Road, Oxford, OX1 3PU (UK)

**Keywords:** fluorescence anisotropy, nanotubes, polycycles, synthetic methods, template synthesis

## Abstract

A π-conjugated twelve-porphyrin tube is synthesized in 32 % yield by a template-directed coupling reaction that joins together six porphyrin dimers, forming twelve new C=C bonds. The nanotube has two bound templates, enclosing an internal volume of approximately 4.5 nm^3^. Its UV/Vis/NIR absorption and fluorescence spectra resemble those of a previously reported six-porphyrin ring, but are red-shifted by approximately 300 cm^−1^, reflecting increased conjugation. Ultrafast fluorescence spectroscopy demonstrates extensive excited-state delocalization. Transfer of electronic excitation from an initially formed state polarized in the direction of the nanotube axis (*z* axis) to an excited state polarized in the *xy* plane occurs within 200 fs, resulting in a negative fluorescence anisotropy on excitation at 742 nm.

The synthesis of π-conjugated belts, barrels, and tubes has become a focus of intense interest.[1–4] Increasing the dimensionality of a π-system is expected to enhance the electronic delocalization,[5] perhaps leading to exotic cooperative electronic phenomena, such as Aharonov–Bohm oscillations,[6,7] which occur in single-walled carbon nanotubes (CNTs), but have not yet been detected in molecular materials. The bottom-up “total synthesis” of structurally defined CNTs or analogous structures could bring many technological benefits, because CNTs have highly desirable optoelectronic properties. However, the current methods for preparing them yield mixtures of species with different chiralities, diameters, and lengths, resulting in broad distributions of properties.[8] Recent efforts towards the rational chemical synthesis of CNTs focused on the use of cycloparaphenylenes as precursors or templates.[1–3,9–11] Herein, we present the synthesis and photophysical behavior of a discrete molecular tube ***t-*****P12** (Figure 1 a), consisting of twelve porphyrins, each of which is directly π-conjugated to its three neighbors. This tube can be viewed as a rim-to-rim dimer of the cyclic porphyrin hexamer ***c-*****P6**, shown as its complex with the **T6** template in Figure 1 b;[12] ***t-*****P12** is also obtained as a template complex, ***t-*****P12⋅(T6)_2_**, with the geometry of a hexagonal prism. A wide variety of synthetic molecular and supramolecular prisms and nanotubes have been reported previously,[13–19] but these structures lacked a π-conjugated cylindrical surface. Cobalt porphyrins have been polymerized on CNT templates to give a material with some similarity to ***t-*****P12**, although that polymer had a poorly defined multilayer structure.[20] The monodisperse tube ***t-*****P12** can be prepared by template-directed synthesis from a readily accessible porphyrin dimer. This work is a significant step towards the bottom-up synthesis of longer well-defined π-conjugated nanotubes.

**Figure 1:**
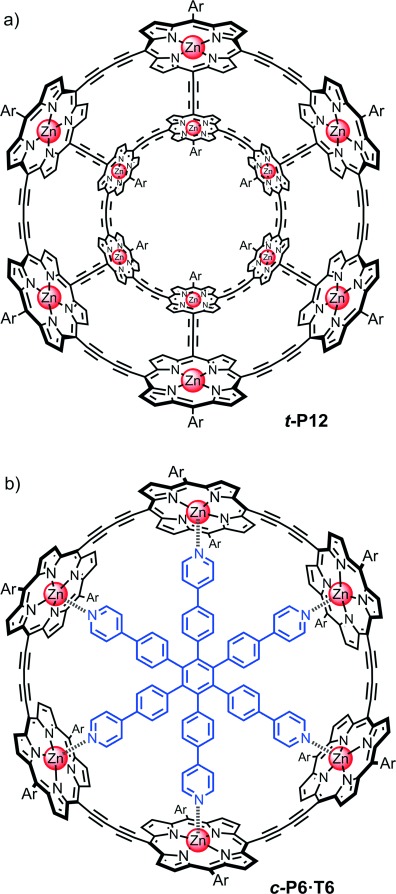
Structures of the twelve-porphyrin tube *t-*P12 (a) and the previously reported six-porphyrin nanoring template complex *c-*P6⋅T6 (b).[12]

The porphyrin nanotube ***t-*****P12⋅(T6)_2_** was synthesized as shown in Scheme 1. The key intermediate is a 5,15-A_2_BC porphyrin, **P1 b**, which was prepared from **P1 a**[21] using a Senge arylation[22] to introduce a bulky solubilizing group, followed by metalation with zinc, bromination at the remaining free meso position, Sonogashira coupling with trimethylsilylacetylene, and selective removal of the TMS group. Palladium-catalyzed homo-coupling of **P1 b** followed by removal of the four triisopropylsilyl groups gave the precursor **P2** for cyclo-oligomerization to form ***t-*****P12⋅(T6)_2_**.

**Scheme 1:**
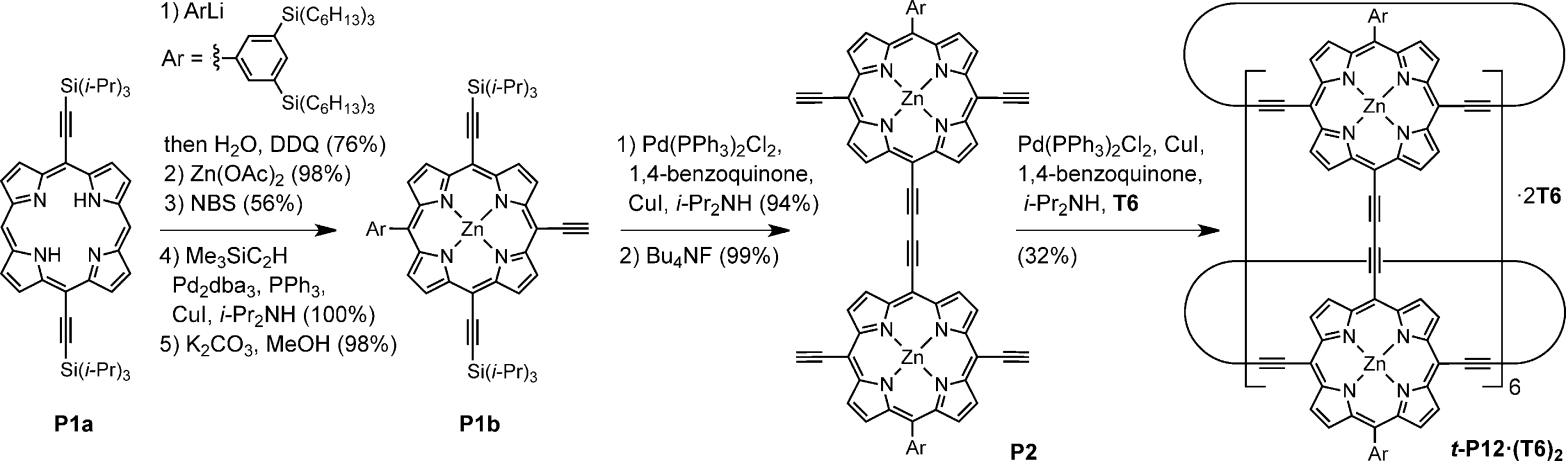
Synthesis of porphyrin nanotube *t-*P12⋅(T6)_2_. dba=dibenzylideneacetone, DDQ=2,3-dichloro-5,6-dicyanobenzoquinone, NBS= *N*-bromosuccinimide.

When we planned the route shown in Scheme 1, it was not clear whether the final step would be feasible. In this step, six molecules of porphyrin dimer **P2** are brought together by two **T6** templates to form ***t-*****P12**. It is known that butadiyne-linked zinc porphyrin dimers such as **P2** bind **T6** to form 3:1 complexes of the type **(P2)_3_⋅T6**,[12] which would have the wrong spatial arrangement to form ***t-*****P12** (Scheme 2). The 6:2 complex, **(P2)_6_⋅(T6)_2_**, with the correct arrangement for tube formation is entropically disfavored relative to **(P2)_3_⋅T6**, although repulsion between the bulky aryl substituents may destabilize **(P2)_3_⋅T6** relative to **(P2)_6_⋅(T6)_2_**. We envisioned that coupling of **P2** molecules would give short belt-like oligomers (such as **P6** in Scheme 2), which would bind to the template in the correct orientation to form the ***t-*****P12** nanotube. In practice, this template-directed coupling reaction was remarkably efficient, as is shown by the GPC chromatogram of the crude reaction mixture (Figure 2 a); the desired product is the dominant peak, and ***t-*****P12⋅(T6)_2_** was isolated in 32 % yield, in a reaction involving the formation of twelve new C=C bonds. The 24 trihexylsilyl groups on the rims of the nanotube provide excellent solubility in solvents such as chloroform, and the compound was readily characterized by mass spectrometry (MALDI-TOF) and NMR spectroscopy (Figure 2).

**Figure 2:**
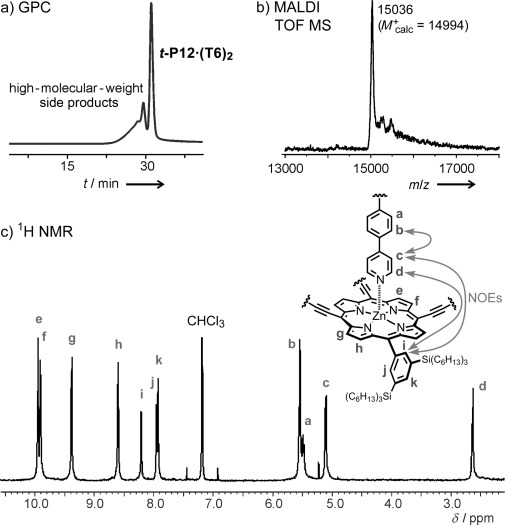
Characterization of *t-*P12⋅(T6)_2_: a) GPC chromatogram (529 nm) of the crude reaction mixture. b) MALDI-TOF spectrum. c) Partial ^1^H NMR spectrum (400 MHz, CDCl_3_) showing selected NOEs.

**Scheme 2:**
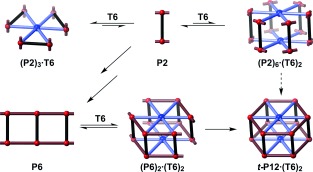
Mechanisms for the formation of *t-*P12⋅(T6)_2_ from dimer P2.

The ^1^H NMR spectrum of ***t-*****P12⋅(T6)_2_** is strikingly simple for a molecule of its size owing to the *D*_6*h*_ symmetry. The assignment of the aromatic protons shown in Figure 2 c is supported by COSY and NOESY spectra (see the Supporting Information), including the observation of NOEs from proton d of the template to protons i, g, and h of the porphyrin. The signals from the template are strongly shielded by the porphyrin ring currents. There are two signals for the protons of the aryl side groups that are *ortho* to the porphyrin (proton i and j, Figure 2 c) because the inside and the outside of the tube provide different environments. The NMR data are consistent with the geometry of ***t-*****P12⋅(T6)_2_** from molecular mechanics calculations (Figure 3 a), and the dimensions of the tube can be compared with crystal structures of its components.[12b,^23^] The Zn–Zn diameter of the tube is 2.43 nm, and the distance between the two **T6** templates is 1.35 Å, resulting in an internal cavity volume of approximately 4.5 nm^3^. The length of the nanotube is 2.3 nm measured to the van der Waals surfaces of the outer β-pyrrole hydrogen atoms, or 3.2 nm measured to the *para* hydrogen atoms of the aryl groups. Although the length of ***t-*****P12** is very short when compared with a CNT, to the best of our knowledge, it is longer than all previously synthesized π-conjugated molecular belts, barrels, or nanotubes.[1–4]

**Figure 3:**
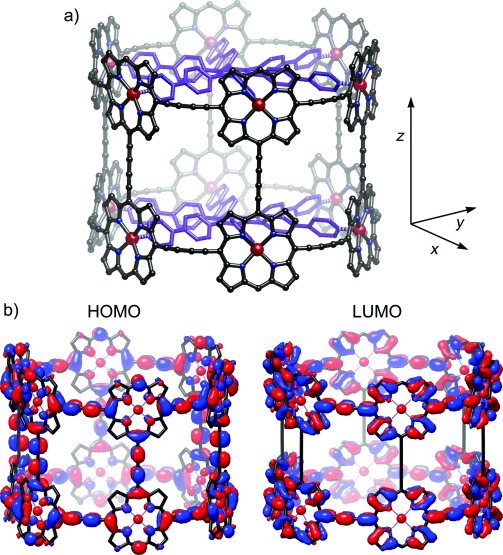
a) Calculated structure of *t-*P12⋅(T6)_2_ with hydrogen atoms and aryl groups bearing solubilizing side chains omitted (modified MM2 force field; see the Supporting Information for details), showing the axes used to discuss the fluorescence anisotropy. b) HOMO and LUMO of *t-*P12 calculated at the BLYP/6-31G(d) level of theory shown with a density isovalue of 0.008. (Aryl groups were omitted to simplify these calculations.)

The UV/Vis/NIR absorption and fluorescence spectra of ***t-*****P12⋅(T6)_2_** are compared with those of ***c-*****P6⋅T6** in Figure 4. Both compounds show similar split absorption and emission bands, indicating that they have similar electronic structures, with emission from a dipole-forbidden first excited state becoming partially allowed through Herzberg–Teller coupling with higher excited states.[12b The absorption and emission maxima of ***t-*****P12⋅(T6)_2_** are red-shifted by approximately 300 cm^−1^ relative to those of ***c-*****P6⋅T6**, reflecting the greater conjugation in the larger π-system. DFT calculations (BLYP/6-31G(d)) indicate that the HOMO of ***t-*****P12⋅(T6)_2_** is distributed over the entire π-system (Figure 3 b) whereas the LUMO is spread over both rings, with negligible coefficients on the butadiyne “staves” connecting the two rings in the *z* direction. Comparison of these frontier orbitals with the four Gouterman orbitals of a simple porphyrin unit shows that the HOMO and LUMO of ***t-*****P12** are derived from the a_2u_ and e_gx_ orbitals of the monomer, respectively,[24] which explains the presence of a node along each stave in the LUMO.

**Figure 4:**
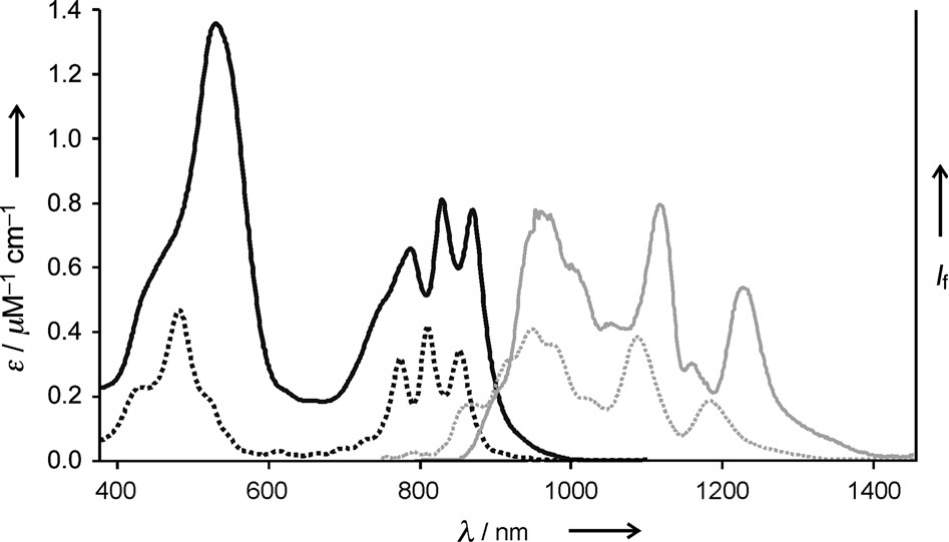
UV/Vis/NIR absorption (black) and fluorescence (gray) spectra of *t-*P12⋅(T6)_2_ (continuous line) and *c-*P6⋅T6 (dashed) recorded in chloroform.

Ultrafast time-resolved fluorescence spectroscopy reveals strong coupling between the components of the nanotube. Figure 5 a shows the fluorescence dynamics of ***t-*****P12⋅(T6)_2_**, excited at 830 nm and 742 nm, with excitation polarized perpendicular or parallel to the detection polarization. The time-dependent fluorescence anisotropies (γ) at these two wavelengths, derived from the data in Figure 5 a, show no change over 0–15 ps after excitation (Figure 5 b). The average anisotropy on excitation at 830 nm is 0.07, which is similar to that recorded for ***c-*****P6⋅T6**[12b,^25^] and close to the theoretical value of 0.1 for an excited state delocalized over a two-dimensional ring.[26] This indicates that in ***t-*****P12⋅(T6)_2_**, absorption at 830 nm is associated with transition dipoles in the plane of the six-porphyrin rings (*xy* plane, Figure 3), and that emission occurs from an excited state that is delocalized over this same *xy* plane. However, at shorter excitation wavelengths, the anisotropy decreases, becoming negative and reaching a value of −0.04 at 742 nm, whereas the fluorescence anisotropy of ***c-*****P6⋅T6** is independent of the wavelength (Figure 5 c). The most likely explanation for this behavior is that at 742 nm, light is absorbed by transitions associated with the *z*-polarized butadiyne-linked porphyrin dimer staves of the barrel to former higher excited states that emit from the *xy*-polarized state. This interpretation is supported by the observation that porphyrin dimer **5 a** has a Q band at 705 nm and by the report that a related square butadiyne-linked porphyrin tetramer exhibits a dimer-like absorption spectrum (Q band at 659 nm).[27] If the absorption by ***t-*****P12⋅(T6)_2_** at 742 nm were entirely *z*-polarized, with emission entirely from a state polarized in the *xy* plane, then the anisotropy would be −0.2.[28] The value of −0.04 reflects the presence of overlapping bands at 742 nm, with different polarizations. Migration of electronic excitation from the *z*-polarized staves to the *xy*-polarized state takes place faster than the 200 fs time resolution of our experiment.

**Figure 5:**
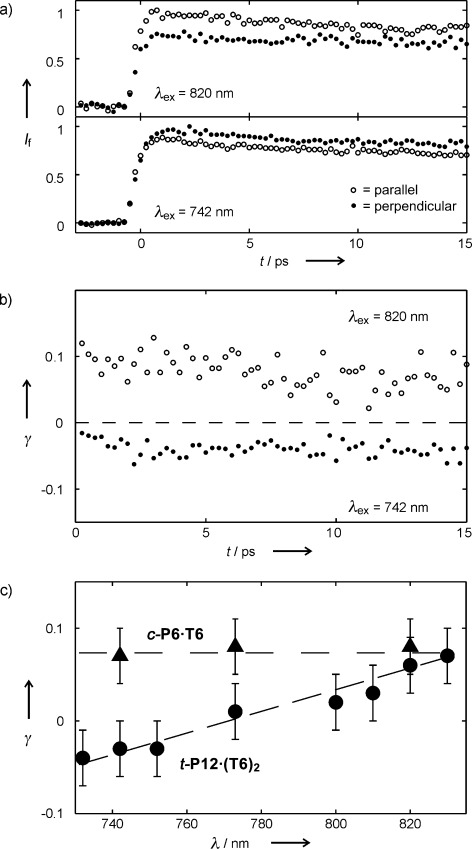
a) Time-resolved fluorescence decay for *t-*P12⋅(T6)_2_ on excitation at 820 nm and 742 nm. Samples are excited by a pulse polarized either parallel (○) or perpendicular (•) to the detection polarization, with detection at 941 nm. b) Fluorescence anisotropy dynamics for excitation at 820 nm (○) and 742 nm (•), calculated from the data in (a). c) Fluorescence anisotropy for *t-*P12⋅(T6)_2_ and *c-*P6⋅T6 measured over a range of excitation wavelengths (with dashed lines as guides to the eye).

In summary, we have synthesized a fully π-conjugated three-dimensional porphyrin nanotube in 32 % yield by an alkyne homo-coupling reaction involving the formation of twelve new C=C bonds. The preparation of this nanotube represents a surprising new type of cooperative template-directed synthesis, in which the binding mode of the templates switches as the reaction progresses; this contrasts with other types of cooperative template-directed syntheses in which the templates assemble the starting materials in the correct geometry for product formation.[29] The absorption and emission spectra of the nanotube are red-shifted by approximately 300 cm^−1^ compared to the corresponding six-porphyrin nanoring. Time-resolved fluorescence anisotropy measurements show that there is ultra-fast energy migration from excited states associated with the staves of the barrel to the six-porphyrin ring regions. Further studies of the electronic delocalization in the radical cations of these molecules are currently underway. We are also investigating the possibility of preparing longer nanotubes using this synthetic strategy.
