# Supplementary material for: A Molecular Nanotube with Three-Dimensional π-Conjugation
Source: Angew Chem Int Ed Engl. 2015 May 7;54(25):7344–8. doi: 10.1002/anie.201502735 (PMC4510782; doi:10.1002/anie.201502735)
Supplement: Supplementary file 1 [file anie0054-7344-sd1.pdf]

Angewandte Chemie
Eine Zeitschrift der Gesellschaft Deutscher Chemiker

Supporting Information

A Molecular Nanotube with Three-Dimensional π-Conjugation**

*Patrik Neuhaus, Arjen Cnossen, Juliane Q. Gong, Laura M. Herz, and Harry L. Anderson**

anie_201502735_sm_miscellaneous_information.pdf

Contents

Synthesis S2

General S2

Free-base porphyrin **1** S2

Zinc porphyrin **2** S3

Bromine-substituted porphyrin **3** S3

Tris alkyne-substituted porphyrin **4** S3

Free alkyne **P1b** S4

Dimer **5** S4

Deprotected dimer **P2** and ***t*-P12·(T6)$_2$** S4

NMR Spectra S5

Compound **1** S5

Compound **2** S6

Compound **3** S7

Compound **4** S8

Compound **P1b** S9

Compound **5** and **P2** S10

***t*-P12·(T6)$_2$** S11

Computational Chemistry S13

Methods S13

Computed Cartesian coordinates and energies S13

Herzberg-Teller vibration S21

Photoluminescence Upconversion Spectroscopy S22

References S22

Synthesis

General

Unless stated otherwise, all reagents were obtained from commercial sources and used as received without further purification. Diethyl ether and toluene were dried by passing over activated alumina. Diisopropylamine was dried by distillation over CaH_2. *N*-Bromosuccinimide was recrystallized from boiling water. 3,5-Trihexylsilylbromobenzene,[S1] hexakis-(4-[4-phenylpyridine])benzene[S2] (**T6**) and 5,15-bis[(triisopropylsilyl)ethynyl]porphyrin[S3] (**P1a**) were synthesized according to literature procedures.

NMR spectra were recorded on a Bruker AVII400, Bruker AVIII400 (400 MHz) or Bruker DRX500 (500 MHz) spectrometer. The residual solvent peak was used as internal reference (chloroform, ^{1}H δ = 7.26 ppm, ^{13}C δ = 77.2 ppm. Multiplicity (s = singlet, d = doublet, t = triplet, q = quartet, and m = multiplet) and coupling constant(s) were reported whenever possible

Analytical gel permeation chromatography (GPC) was performed on a JAIGEL H-P pre-column, a JAIGEL 3H-A (8 mm × 500 mm) and a JAIGEL 4H-A column (8 mm × 500 mm) in series with toluene/pyridine 100/1 as eluent or on two PLgel 3 µm Mixed-E columns (2 × 300 mm length, 7.5 mm diameter) and two PLgel 5 µm Mixed-D columns (3 × 300 mm length, 7.5 mm diameter) in series with THF as an eluent. Preparative recycling GPC was performed on a JAIGEL H-P pre-column, a JAIGEL 3H (20 mm × 600 mm) and a JAIGEL 4H column (20 mm × 600 mm) in series with toluene/pyridine 100/1 as eluent.

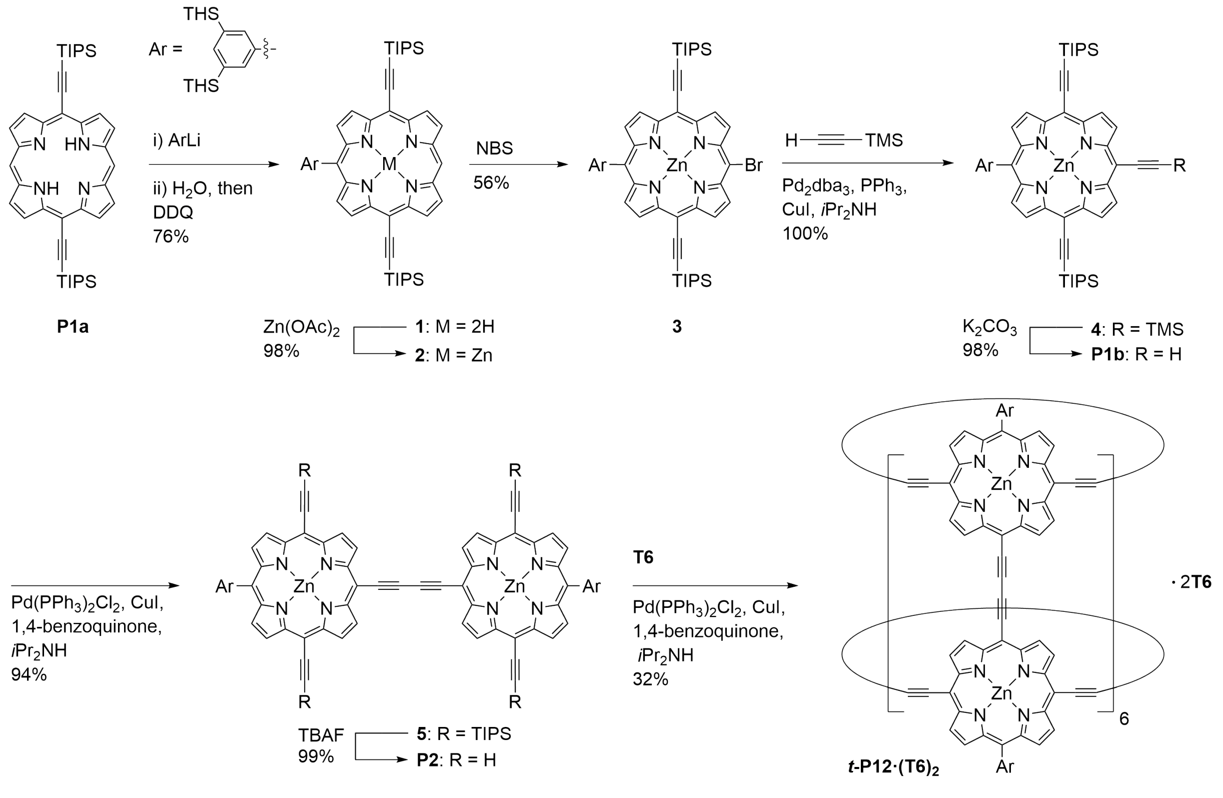

Figure S1. Synthesis of nanotube ***t*-P12·(T6)$_2$.**

Free-base porphyrin 1

To a solution of 3,5-trihexylsilylbromobenzene (0.65 g, 0.90 mmol) in Et_2O (5 mL) at –78 °C under N_2 atmosphere was added dropwise *n*-BuLi (1.6 M in hexanes, 0.56 mL, 0.90 mmol). The mixture was warmed to room temperature and stirred for 1 h. This solution was added dropwise to a solution of **P1a** (100 mg, 0.15 mmol) in THF (5 mL). The mixture was stirred at room temperature overnight. Water (0.5 mL) was added, followed by DDQ (102 mg, 0.45 mmol). The reaction mixture was stirred

for 20 min before addition of Et_3N (0.2 mL). The volatiles were removed *in vacuo* and the residue was taken up in CH_2Cl_2 (50 mL) and washed with H_2O (2 × 30 mL). The organic layer was dried over Na_2SO_4 and concentrated. Column chromatography (9:1 PE:CH_2Cl_2) afforded **1** (0.15 g, 0.11 mmol, 76%) as a purple solid. **^{1}H NMR ($CDCl_3$, 400 MHz)**: 10.06 (s, 1H), 9.75 (d, *J* = 4.4 Hz, 2H), 9.68 (d, *J* = 4.8 Hz, 2H), 9.29 (d, *J* = 4.4 Hz, 2H), 8.85 (d, *J* = 4.8 Hz, 2H), 8.25 (s, 2H), 8.02 (s, 1H), 1.55–1.45 (54H), 1.45–1.36 (m, 12H), 1.36–1.29 (m, 24H), 0.98–0.92 (m, 12H), 0.92–0.87 (m, 18H), –2.36 (s, 2H). **^{13}C NMR ($CDCl_3$, 125 MHz)**: 149.4 (4C α-pyrrole), 146.4 (4C α-pyrrole), 140.4 (2CH *o*-Ar), 140.2 (CH *p*-Ar), 139.7 (C *ipso*-Ar), 135.2 (2C *m*-Ar), 132.4 (2C β-pyrrole), 131.7 (2C β-pyrrole), 130.9 (2C β-pyrrole), 130.2 (2C β-pyrrole), 123.7 (C *meso*), 108.6 (CH *meso*), 106.3 (2C *meso*), 100.8 (2C C≡C), 99.5 (2C C≡C), 33.7 (6CH_2 $Si(CH_3)_3$), 31.8 (6CH_2 $Si(CH_3)_3$), 24.3 (6CH_2 $Si(CH_3)_3$), 22.8 (6CH_2 $Si(CH_3)_3$), 19.2 (12CH_3 TIPS), 14.3 (6CH_3 $Si(CH_3)_3$), 12.9 (6CH TIPS), 12.1 (6CH_2 $Si(CH_3)_3$). **MS (MALDI)** calcd. for $C_{84}H_{134}N_4Si_4Zn$: 1310.97, found: 1311.16.

Zinc porphyrin 2

To a solution of **1** (0.15 g, 0.11 mmol) in $CHCl_3$ (5 mL) was added a solution of zinc acetate dihydrate (0.11 g, 0.50 mmol) in MeOH (1 mL). The mixture was stirred at room temperature for 1 h before filtration over a short silica plug (100:1 CH_2Cl_2:pyridine). Compound **2** (1:1 complex with pyridine, 164 mg, 98%) was obtained as a purple solid. **^{1}H NMR ($CDCl_3$, 400 MHz)**: 10.04 (s, 1H), 9.81 (d, *J* = 4.4 Hz, 2H), 9.73 (d, *J* = 4.4 Hz, 2H), 9.31 (d, *J* = 4.0 Hz, 2H), 8.87 (d, *J* = 4.4 Hz, 2H), 8.23 (s, 2H), 7.99 (s, 1H), 1.55–1.44 (54H), 1.44–1.35 (m, 12H), 1.35–1.29 (m, 24H), 0.97–0.91 (m, 12H), 0.91–0.86 (m, 18H). **^{13}C NMR ($CDCl_3$, 100 MHz)**: 152.6 (2C), 152.4 (2C), 150.2 (2C), 150.0 (2C), 141.2 (C), 140.4 (2CH), 139.2 (CH), 134.6 (2C), 132.8 (2CH), 132.2 (2CH), 131.6 (2CH), 130.8 (2CH), 124.2 (C), 110.4 (CH), 107.1 (2C), 100.6 (2C), 97.3 (2C), 33.7 (6CH_2), 31.8 (6CH_2), 24.2 (6CH_2), 22.8 (6CH_2), 19.3 (12CH_3), 14.3 (6CH_3), 12.9 (6CH), 12.1 (6CH_2). **MS (MALDI)** calcd. for $C_{84}H_{132}N_4Si_4Zn$: 1372.88, found: 1373.26.

Bromide-substituted porphyrin 3

To a solution of **2** (164 mg, 0.11 mmol) in $CHCl_3$ (5 mL) and pyridine (0.5 mL) was added *N*-bromosuccinimide (21 mg, 0.12 mmol). The mixture was stirred overnight at room temperature. TLC (4:1 petrol:CH_2Cl_2, $R_f \approx 0.45$, starting material is fluorescent while product is not) indicated the presence of starting material and additional NBS (2 × 10 mg) was added until the reaction was complete. The volatiles were removed *in vacuo* and the residue was purified by column chromatography (4:1 PE:CH_2Cl_2, + 1% pyridine) yielding **3** (97 mg, 0.067 mmol, 56%) as purple solid. **^{1}H NMR ($CDCl_3$, 400 MHz)**: 9.77 (d, *J* = 4.4 Hz, 2H), 9.71 (d, *J* = 4.4 Hz, 2H), 9.69 (d, *J* = 4.4 Hz, 2H), 8.84 (d, *J* = 4.4 Hz, 2H), 8.22 (s, 2H), 8.02 (s, 1H), 1.59–1.47 (54H), 1.47–1.38 (m, 12H), 1.38–1.32 (m, 24H), 1.02–0.95 (m, 12H), 0.95–0.90 (m, 18H).**^{13}C NMR (CDCl3, 125 MHz)**: 152.8 (2C), 152.7 (2C), 151.0 (2C), 149.6 (2C), 140.7 (C), 140.2 (2CH), 139.2 (CH), 134.8 (2C), 133.3 (2C), 133.1 (2C), 132.2 (2C), 131.3 (2C), 124.5 (C), 109.8 (C), 106.0 (2C), 101.9 (2C), 98.2 (2C), 33.7 (6CH_2), 31.7 (6CH_2), 24.1 (6CH_2), 22.7 (6CH_2), 19.2 (12CH_3), 14.3 (6CH_3), 12.8 (6CH), 12.1 (6CH_2). **MS (MALDI)** calcd. for $C_{84}H_{13}BrN_4Si_4Zn$: 1453.79, found: 1453.12.

Tris alkyne-substituted porphyrin 4

In a 25-mL two-necked flask were placed **3** (97 mg, 0.067 mmol), Pd_2dba_3 (6.4 mg, 0.007 mmol), CuI (1.3 mg, 0.007 mmol) and triphenylphosphine (3.7 mg, 0.014 mmol). Toluene (4 mL) and *N*,*N*-diisopropylamine (2 mL) were added and the mixture was deoxygenated by freeze-pump-thaw cycles. Trimethylsilylacetylene (14 µL, 0.1 mmol) was added and the reaction mixture was stirred overnight at room temperature. The volatiles were removed *in vacuo* and the residue was purified by column chromatography (5:1 PE:CH_2Cl_2, + 1% pyridine) yielding **4** (98 mg, 0.067 mmol, 100%) as a purple solid. **^{1}H NMR ($CDCl_3$, 400 MHz)**: 9.76 (d, *J* = 4.4 Hz, 2H), 9.71 (d, *J* = 4.4 Hz, 2H), 9.65 (d, *J* = 4.4 Hz, 2H), 8.80 (d, *J* = 4.4 Hz, 2H), 8.21 (s, 2H), 8.01 (s, 1H), 1.59–1.46 (54H), 1.47–1.38 (m, 12H), 1.38–1.30 (m, 24H), 1.00–0.94 (m, 12H), 0.94–0.89 (m, 18H), 0.69 (s, 9H). **^{13}C NMR ($CDCl_3$, 100 MHz)**: 152.6 (2C), 152.5 (2C), 152.3 (2C), 150.1 (2C), 140.7 (C), 140.1 (2CH), 139.1 (CH), 134.7 (2C), 132.9 (2CH), 131.8 (2CH), 131.5 (2CH), 130.6 (2CH), 125.6 (C), 109.8 (C), 108.0 (2C), 102.0 (2C), 100.6 (C), 100.3 (C), 97.8 (2C), 33.5 (6CH_2), 31.6 (6CH_2), 24.1 (6CH_2), 22.6 (6CH_2), 19.1

(12CH$_3$), 14.2 (6CH$_3$), 12.7 (6CH), 12.0 (6CH$_2$), 0.5 (3CH$_3$). **MS (MALDI)** calcd. for $C_{89}H_{140}N_4Si_5Zn$: 1469.92, found: 1467.8.

Free alkyne P1b

To a solution of **4** (280 mg, 0.190 mmol) in THF (12 mL) and MeOH (12 mL) was added K_2CO_3 (262 mg, 1.90 mmol). The suspension was stirred at 20 °C for 30 min before removal of the volatiles *in vacuo*. The residue was taken up in CH_2Cl_2 (30 mL) and washed with H_2O (2 × 30 mL). The organic layer was dried over Na_2SO_4 and concentrated. The residue was subjected to column chromatography (6:1 PE:CH_2Cl_2 +1% pyridine) yielding **P1b** (261 mg, 0.186 mmol, 98%) as a green-purple solid. **^{1}H NMR (CDCl$_3$, 400 MHz)**: 9.73 (d, J = 4.6 Hz, 2H), 9.69 (d, J = 4.6 Hz, 2H), 9.62 (d, J = 4.6 Hz, 2H), 8.78 (d, J = 4.6 Hz, 2H), 8.17 (s, 2H), 7.96 (s, 1H), 4.16 (s, 1H), 1.55–1.41 (m, 54H), 1.41–1.33 (m, 12H), 1.33–1.25 (m, 24H), 0.95–0.89 (m, 12H), 0.89–0.83 (m, 18H); **^{13}C NMR (CDCl$_3$, 125 MHz)**: 152.8 (2C), 152.6 (2C), 152.5 (2C), 150.2 (2C), 140.7 (C), 140.2 (2CH), 139.3 (CH), 134.8 (2C), 133.1 (2CH), 132.1 (2CH), 131.5 (2CH), 130.8 (2CH), 125.8 (C), 109.8 (2C), 102.1 (CH), 99.1 (C), 98.1 (C), 86.5 (C), 83.2 (C), 33.7 (6CH$_2$), 31.7 (6CH$_2$), 24.2 (6CH$_2$), 22.8 (6CH$_2$), 19.3 (12CH$_3$), 14.3 (6CH$_3$), 12.8 (6CH), 12.1 (6CH$_2$). **MS (MALDI)** calcd. for $C_{86}H_{132}N_4Si_4Zn$: 1398.88, found: 1397.86.

Dimer 5

To a solution of **P1b** (261 mg, 0.186 mmol) in dry toluene (5 mL) and diisopropylamine (5 mL) was added $Pd(PPh_3)_2Cl_2$ (6.3 mg, 9 µmol), CuI (17 mg, 0.09 mmol) and 1,4-benzoquinone (41 mg, 0.38 mmol). The mixture was stirred at 20 °C for 4 h before removal of the volatiles *in vacuo*. The residue was passed through a short silica column (4:1 PE:CH_2Cl_2 +1% pyridine) and subsequently purified by size-exclusion chromatography (BioBeads SX-1, toluene) yielding **5** (246 mg, 0.088 mmol, 94%) as a green-purple solid. **^{1}H NMR (CDCl$_3$, 400 MHz)**: 9.98 (d, J = 4.6 Hz, 4H), 9.86 (d, J = 4.6 Hz, 4H), 9.64 (d, J = 4.6 Hz, 4H), 8.80 (d, J = 4.6 Hz, 4H), 8.21 (s, 4H), 8.00 (s, 2H), 1.60–1.45 (m, 108H), 1.45–1.36 (m, 24H), 1.36–1.29 (m, 48H), 0.99–0.92 (m, 24H), 0.92–0.87 (m, 36H). **MS (MALDI)** calcd. for $C_{172}H_{262}N_8Si_8Zn_2$: 2796.75, found: 2794.58. **UV/vis/NIR (chloroform)** λ_{max} (log ε): 459 (5.43), 482 (5.32), 514 (5.42), 664 (4.75), 705 (4.85).

Deprotected dimer P2 and *t*-P12·(T6)$_2$

To a solution of **5** (42 mg, 0.015 mmol) in CH_2Cl_2 (8 mL) was added TBAF (0.15 mL of a 1.0 M solution in THF, 0.15 mmol). The solution was stirred at 20 °C for 30 min and then directly passed through a silica plug (CH_2Cl_2 +1% pyridine) yielding crude **P2** (~35 mg) after evaporation. This was used in the next step without further purification. **^{1}H NMR (CDCl$_3$, 400 MHz)**: 9.96 (d, J = 4.6 Hz, 4H), 9.81 (d, J = 4.6 Hz, 4H), 9.61 (d, J = 4.6 Hz, 4H), 8.82 (d, J = 4.6 Hz, 4H), 8.21 (s, 4H), 7.98 (s, 2H), 4.20 (s, 4H), 1.55–143 (m, 24H), 1.42–1.33 (m, 24H), 1.33–1.26 (m, 48H), 0.96–0.90 (m, 24H), 0.90–0.85 (m, 36H). Crude **P2** was dissolved in $CHCl_3$ (~10 mL) and hexakis-(4-[4-phenylpyridine])benzene (**T6**) (23.7 mg, 0.024 mmol) was added. After stirring for 5 min, a UV/vis absorption spectrum was recorded to check formation of the complex. The solution was concentrated and the residue re-dissolved in dry toluene (10 mL) and diisopropylamine (5 mL). The solution was heated to 65 °C and subsequently $Pd(PPh_3)_2Cl_2$ (2.1 mg, 3 µmol), CuI (2.9 mg, 1.5 µmol) and 1,4-benzoquinone (3.2 mg, 0.030 mmol) were added. Heating was continued for 2.5 h, and then the reaction mixture was allowed to cool before loading directly onto a silica plug and eluted with toluene. After removal of the volatiles *in vacuo*, the residue was passed through a second silica plug with CH_2Cl_2. The material was further purified by size-exclusion chromatography (BioBeads SX-1, toluene) and finally subjected to recycling GPC yielding ***t*-P12·(T6)$_2$** (12 mg, 0.8 µmol, 32%) as a purple-pink solid. **^{1}H NMR (CDCl$_3$, 400 MHz)**: 9.95 (d, J = 4.2 Hz, 24H), 9.91 (d, J = 4.2 Hz, 24H), 9.38 (d, J = 4.2 Hz, 24H), 8.60 (d, J = 4.2 Hz, 24H), 8.21 (s, 12H), 7.96 (s, 12H), 7.93 (s, 12H), 5.60–5.45 (m, 48H), 5.11 (d, J = 5.9 Hz, 24H), 2.64 (d, J = 5.9 Hz, 24H), 1.45–1.36 (m, 144H), 1.36–1.29 (m, 144H), 1.29–1.18 (m, 288H), 0.95–0.82 (m, 216H), 0.82–0.71 (m, 144H). **MS (MALDI-TOF)** calculated for $C_{960}H_{1164}N_{60}Si_{24}Zn_{12}$: 14994; found: 15036. **UV/vis/NIR (toluene)** λ_{max} (log ε): 528 (5.89), 791 (5.60), 831 (5.72), 873 (5.71).

NMR Spectra

Compound 1

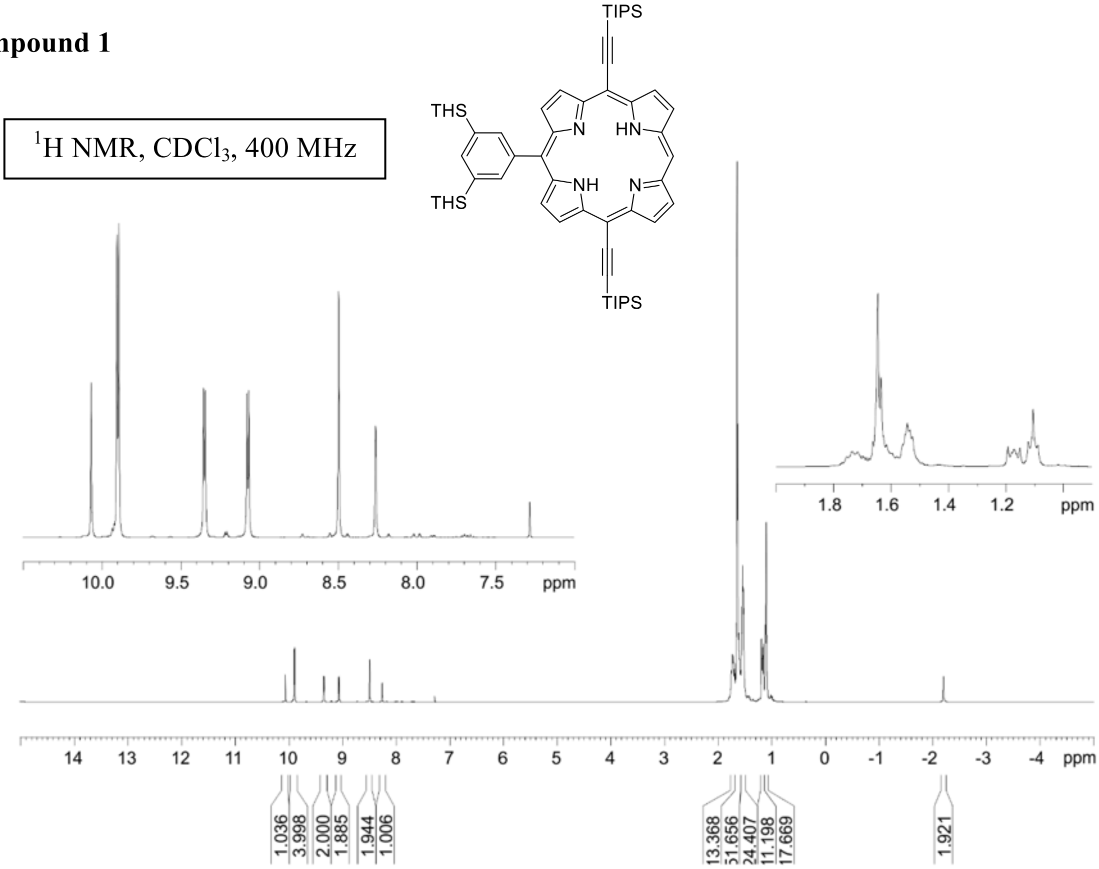

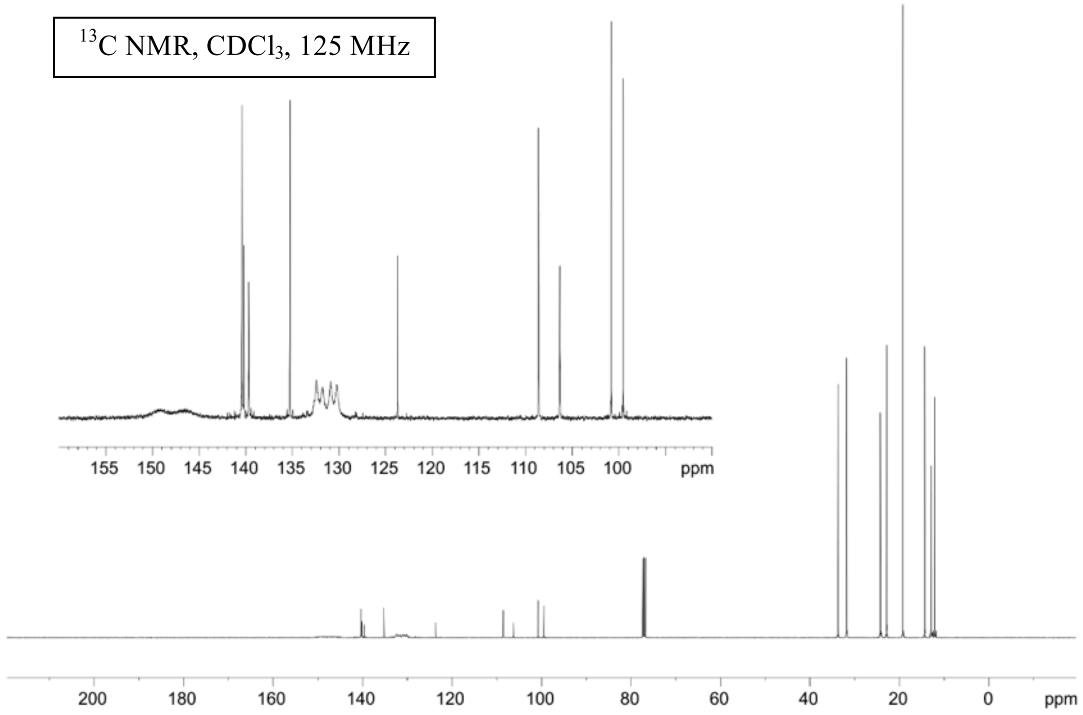

Compound 2

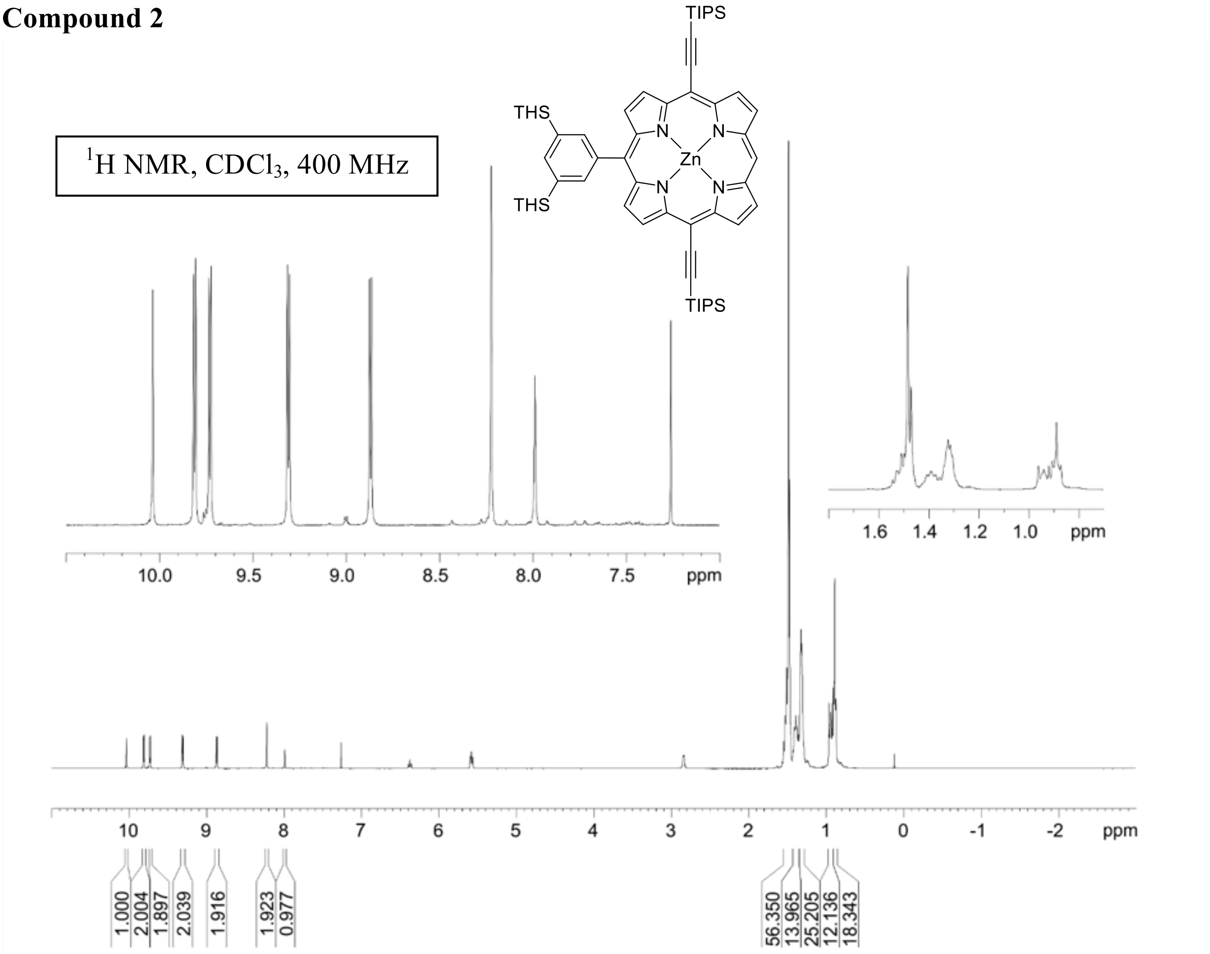

TIPS
THS
THS
N
N
Zn
N
N
TIPS
^{1}H NMR, CDCl$_3$, 400 MHz
ppm
1.000
2.004
1.897
2.039
1.916
1.923
0.977
56.350
13.965
25.205
12.136
18.343

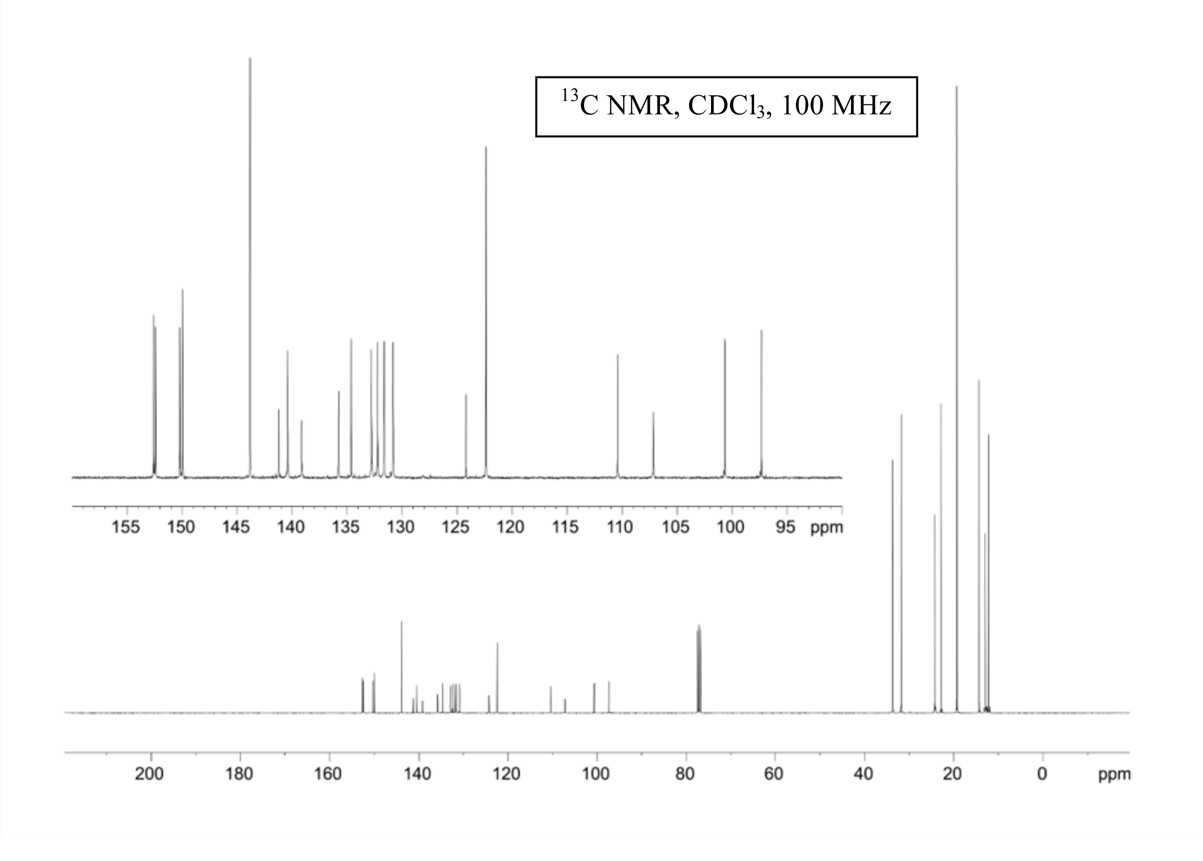

^{13}C NMR, CDCl$_3$, 100 MHz
ppm

Compound 3

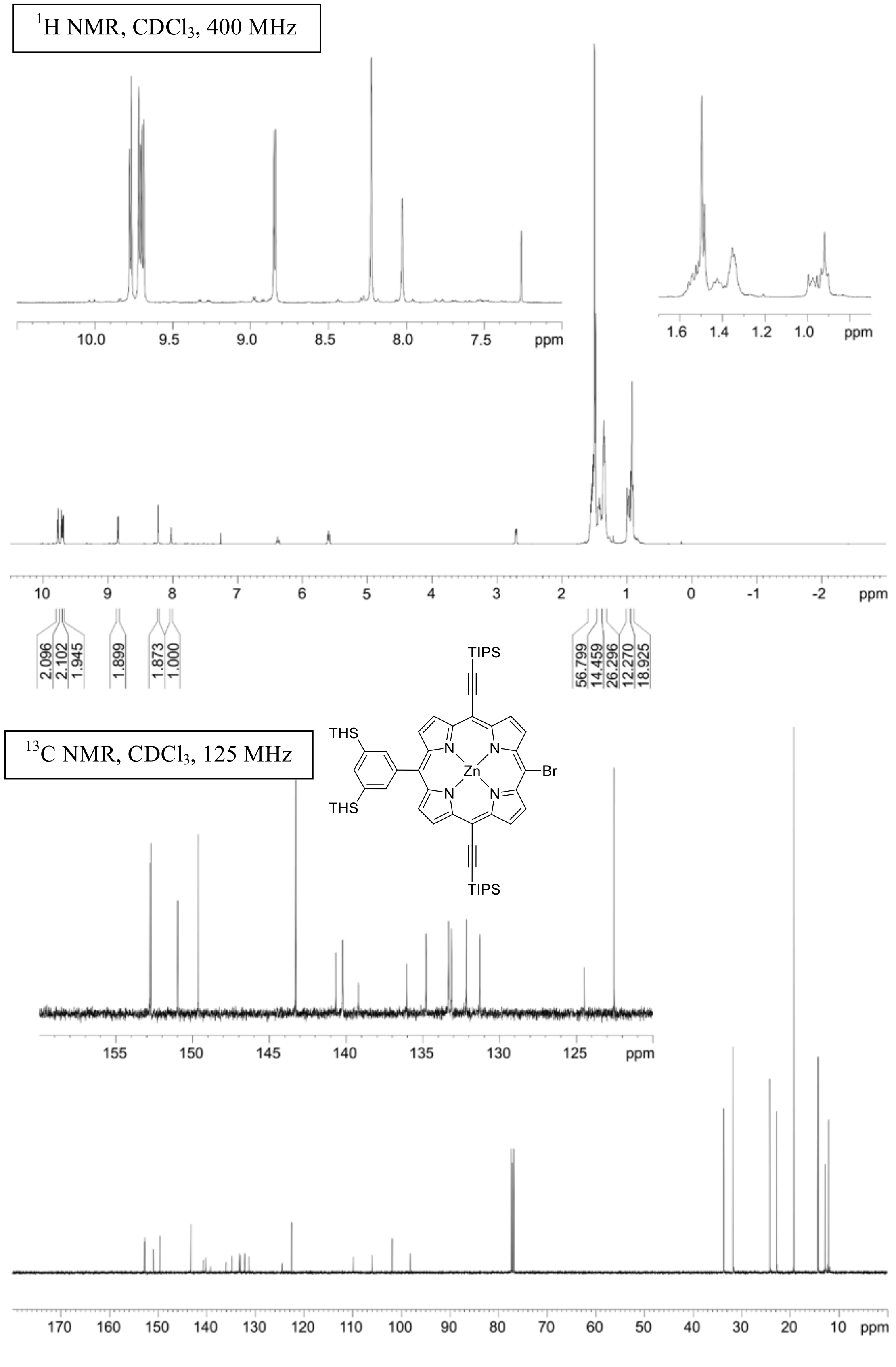
¹H NMR, CDCl₃, 400 MHz
2.096
2.102
1.945
1.899
1.873
1.000
56.799
14.459
26.296
12.270
18.925
ppm
TIPS
THS
THS
Zn
N
Br
TIPS
¹³C NMR, CDCl₃, 125 MHz
ppm

Compound 4

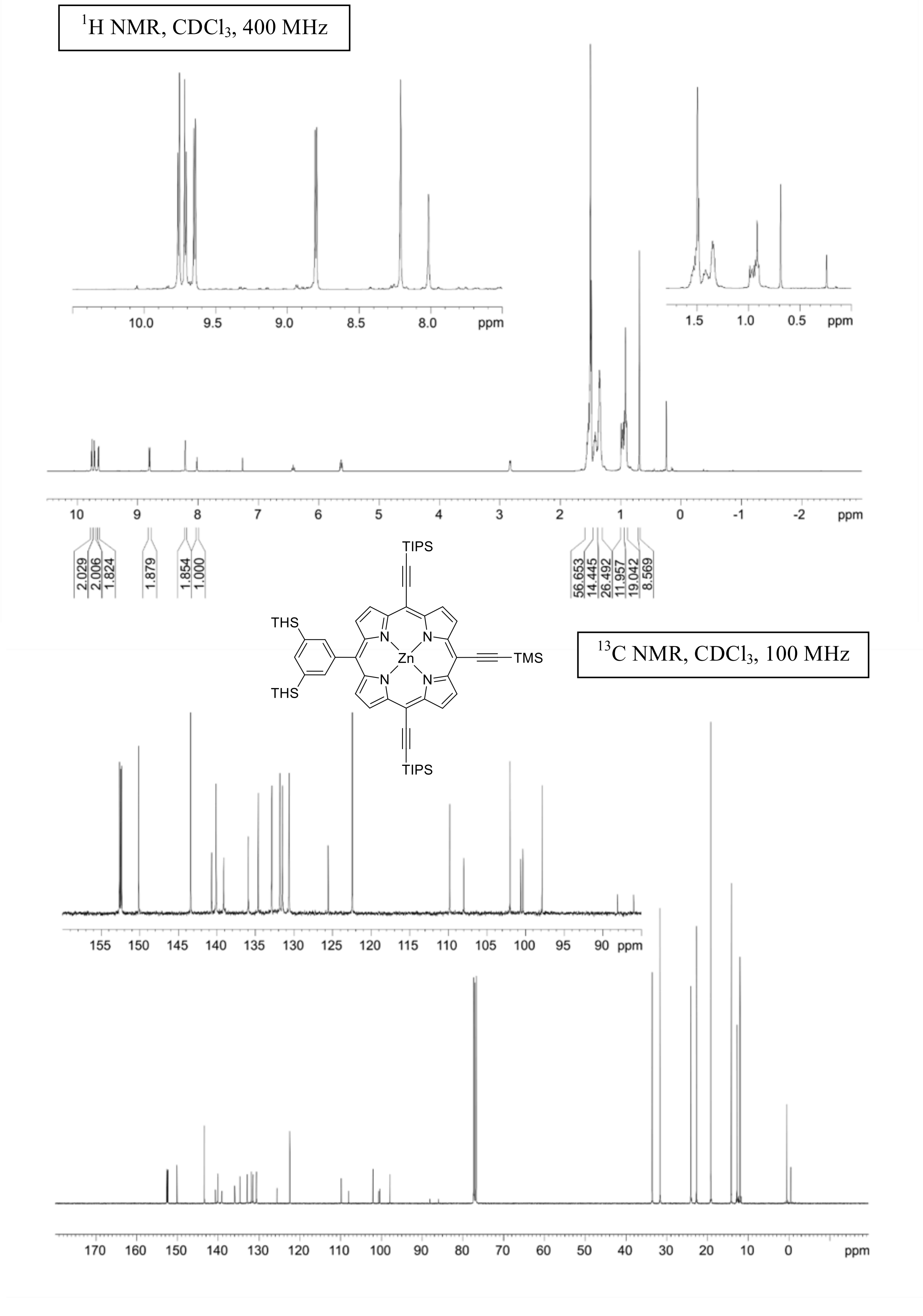

1H NMR, CDCl3, 400 MHz
ppm
2.029
2.006
1.824
1.879
1.854
1.000
56.653
14.445
26.492
11.957
19.042
8.569
TIPS
THS
THS
Zn
TMS
TIPS
13C NMR, CDCl3, 100 MHz
ppm

Compound P1b

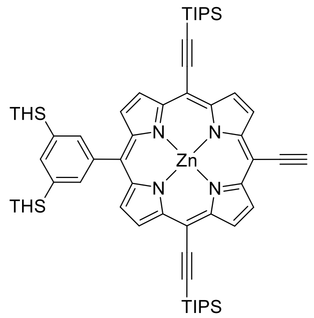

^{1}H NMR, $CDCl_3$, 400 MHz

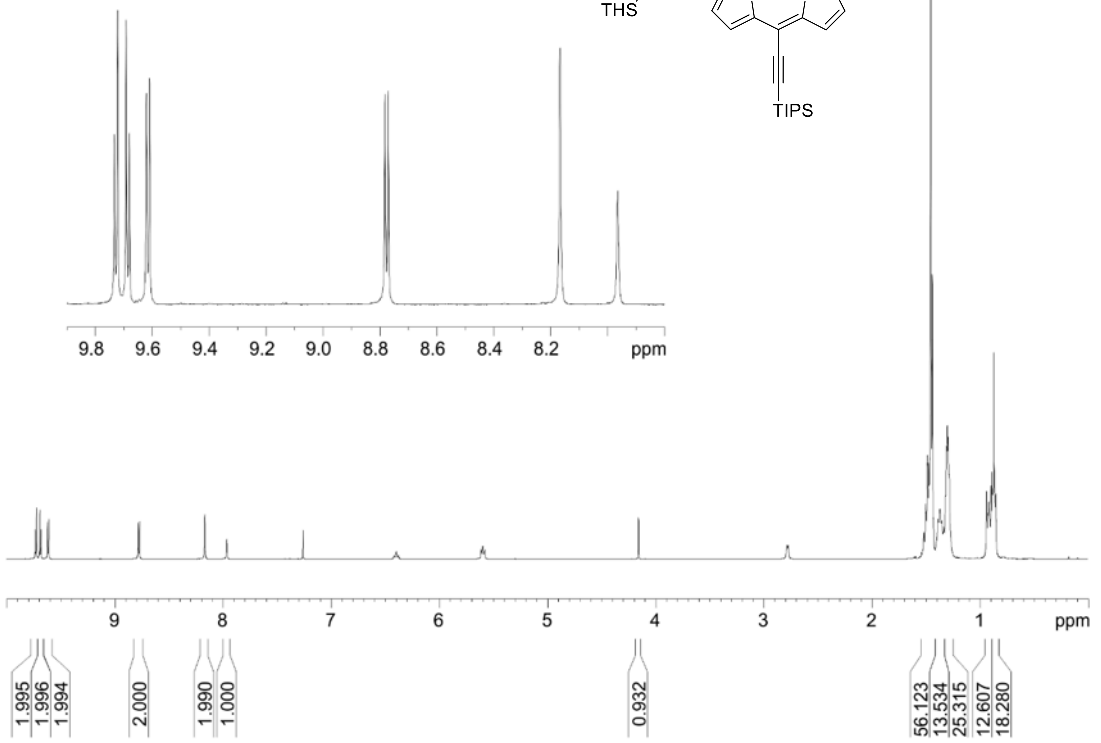

^{13}C NMR, $CDCl_3$, 400 MHz

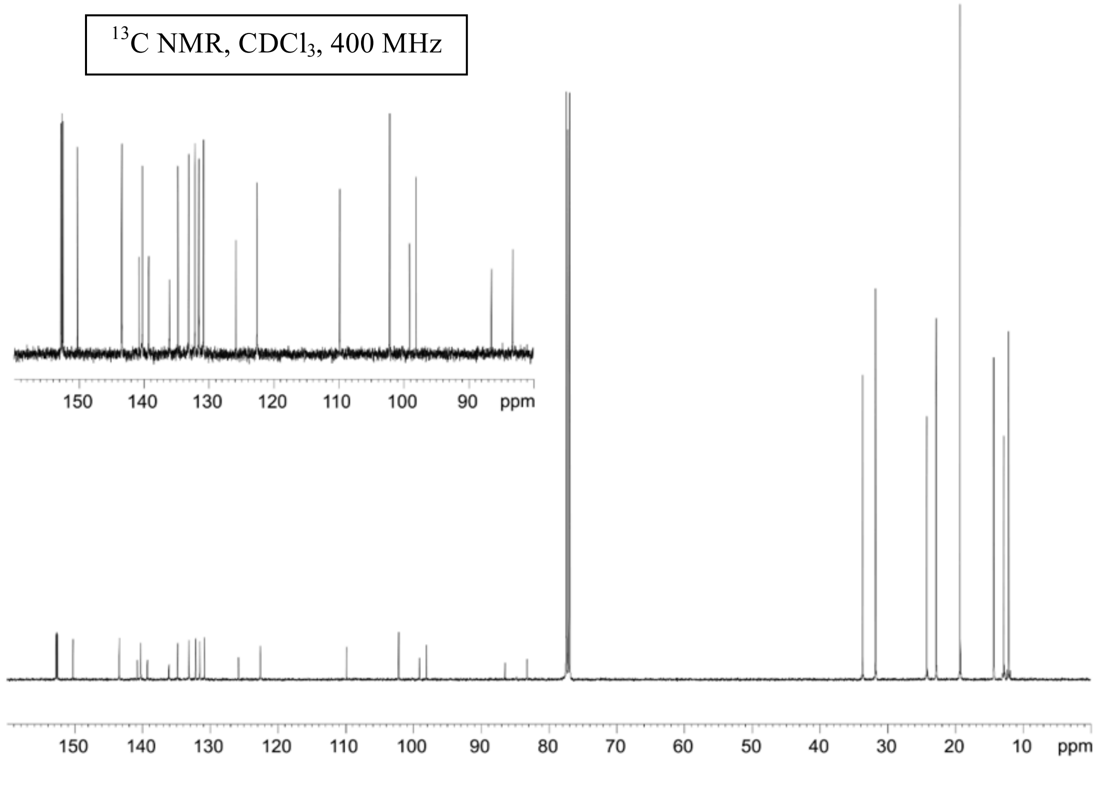

Compound 5 and P2

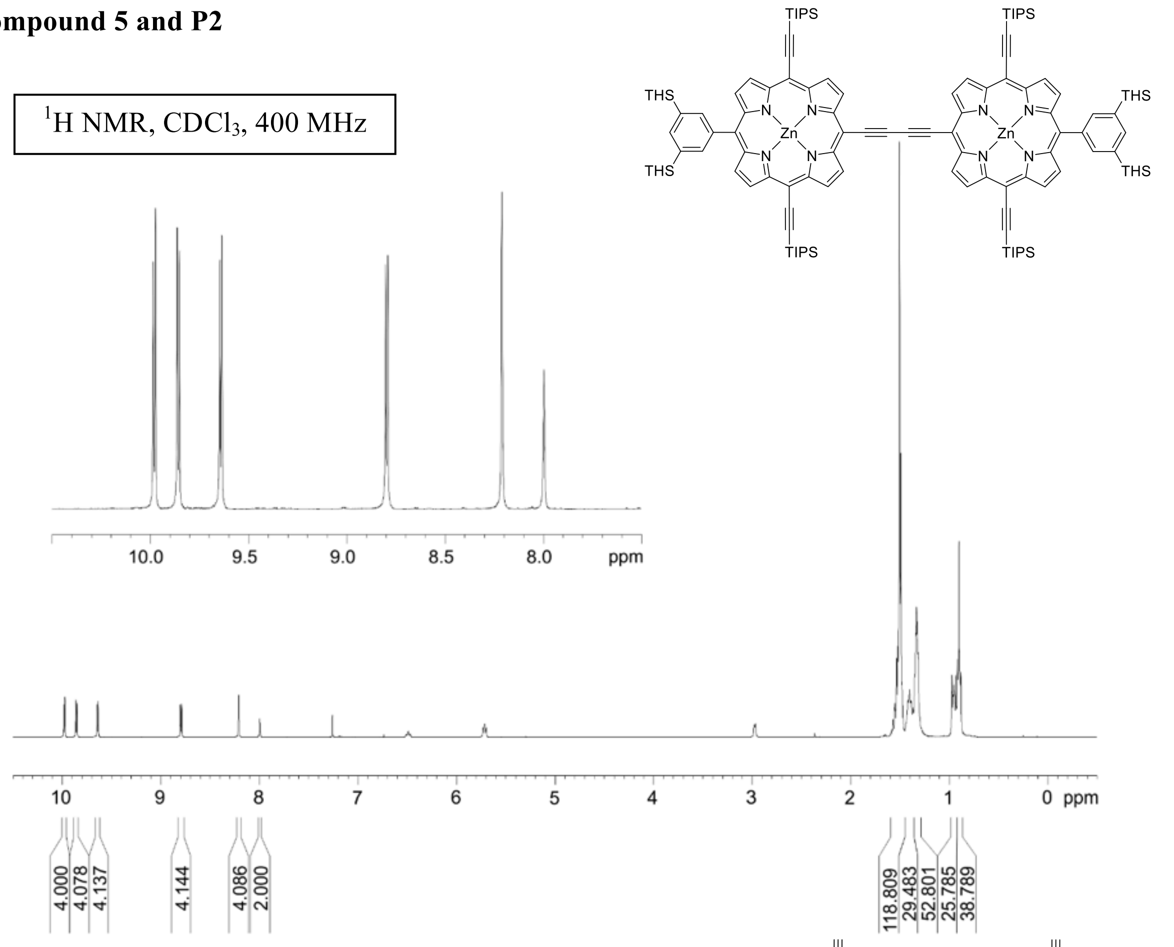

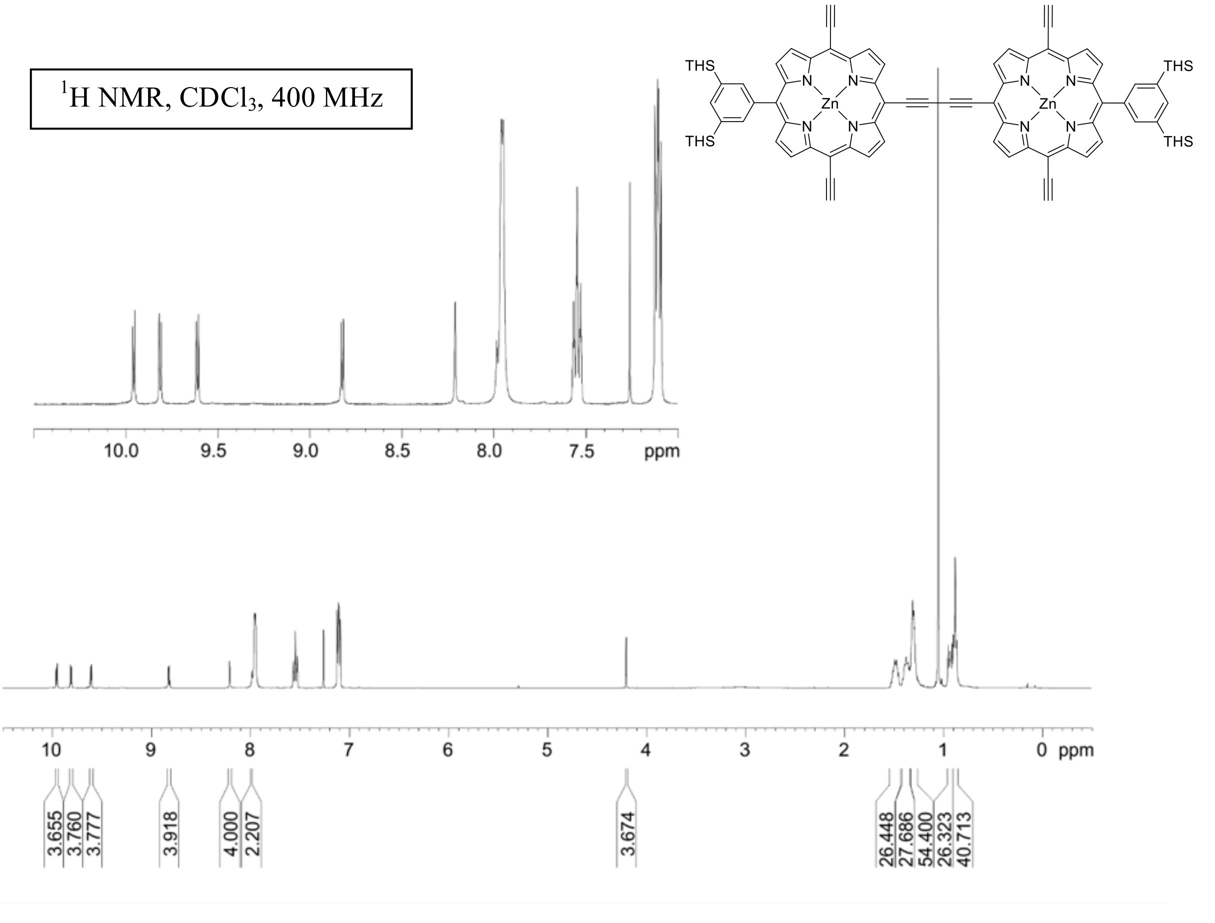

***t*-P12·(T6)$_2$**

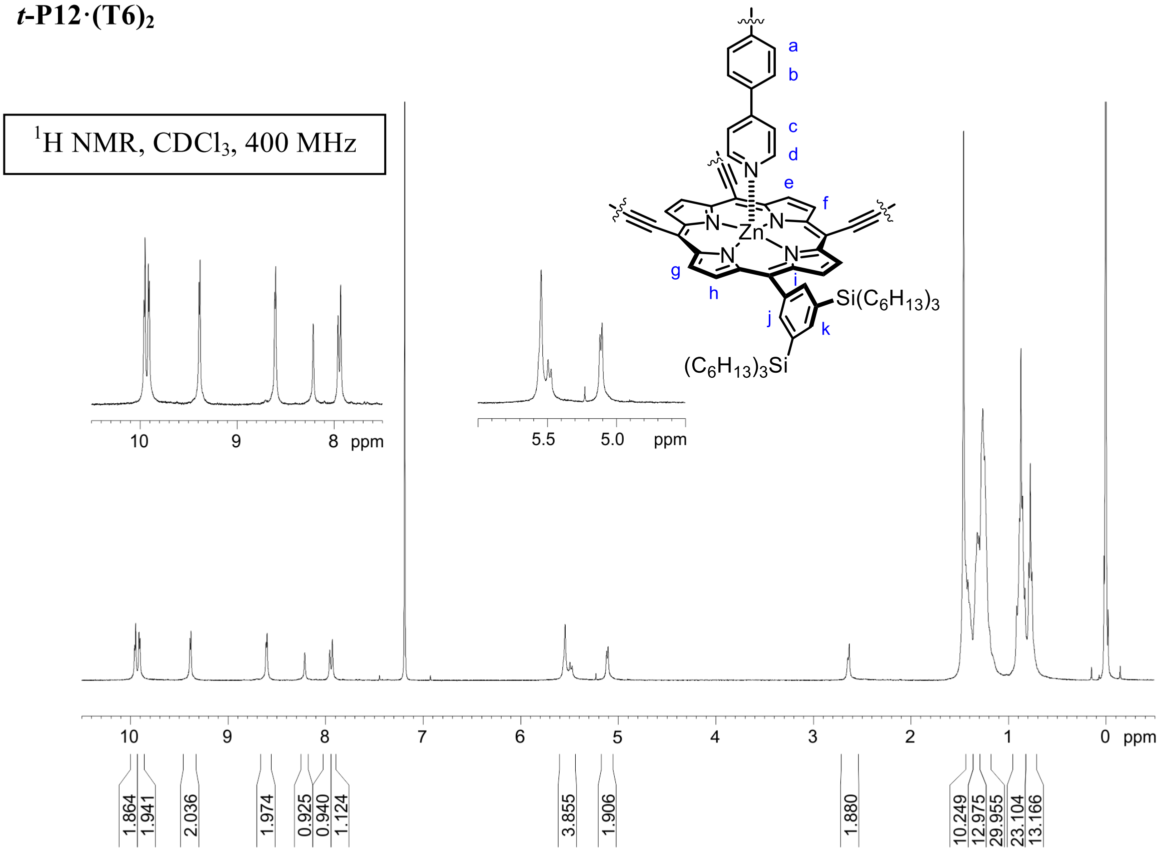

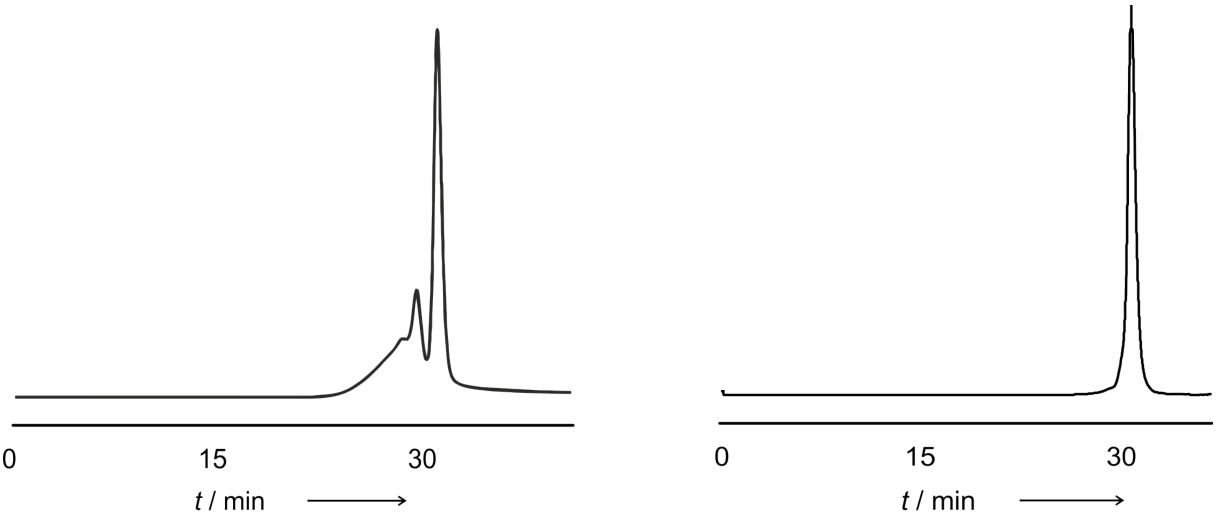

Figure S2. GPC (THF) trace of ***t*-P12·(T6)$_2$** before (left) and after (right) purification by recycling GPC. Detection wavelength: 529 nm. Column: two PLgel 3 μm Mixed-E columns (2 × 300 mm length, 7.5 mm diameter) and two PLgel 5 μm Mixed-D columns (3 × 300 mm length, 7.5 mm diameter) in series.

COSY:

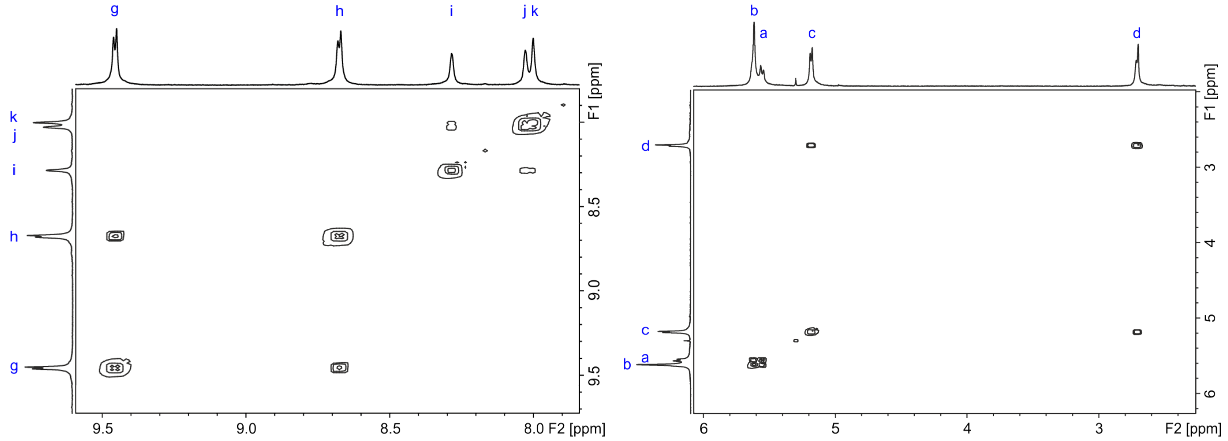

NOESY:

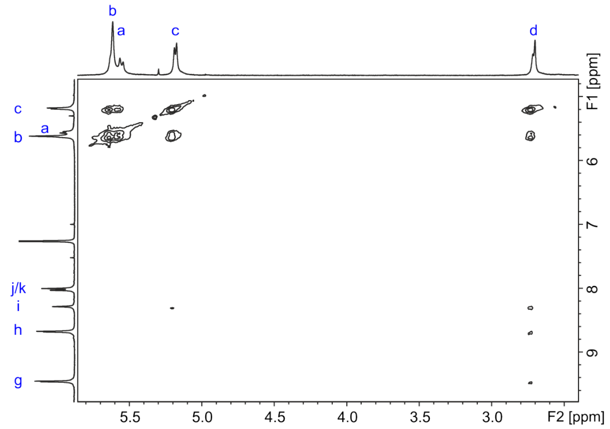

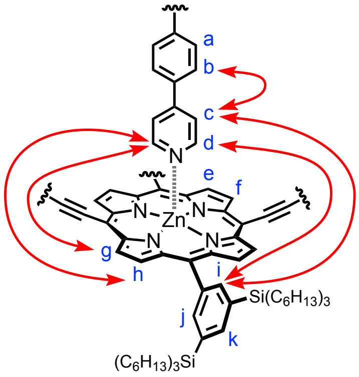

Table S1. Overview of COSY interactions

	a	b	c	d	g	h	i	j	k
a		+							
b	+								
c				+					
d			+						
g						+			
h					+				
i								+	+
j							+		
k							+		

Table S2. Overview of NOESY interactions

	a	b	c	d	g	h	i	j	k
a			s	w					
b			s	s					
c	s	s		s			w		
d	w	s	s		w	w	w		
g				w		s	w	w	
h				w	s		s	s	w
i			w	w	w	s		w	w
j					w	s	w		
k						w	w		

s: strong, w: weak

Computational Chemistry

Methods

Molecular mechanics calculations on the complex ***t*-P12·(T6)$_2$** were performed using HyperChem 8. A modified form of HyperChem's MM+ forcefield was used, to which additional bond stretch, angle bend and torsion terms had been added to describe metalloporphyrins,[S4,S5] alkynes[S6] and butadiynes.[S7] Minimizations were performed using the steepest descent and Polak-Ribiere algorithms.

The optimized geometry and vibrational frequencies of ***t*-P12** were calculated at the restricted BLYP[S8-S10] level of theory employing the 6-31G(d) polarized valence-double-ξ basis set,[S11-S16] neglecting aryl sidegroups and templates using a C1 symmetry of ***t*-P12** within the calculation. Tight convergence criteria were used throughout. No imaginary frequencies were found for the optimized structure. Ground state vibrational frequencies of the butadiyne stretching vibrations are reported to draw a comparison to the HT active modes observed in the fluorescence spectra. All DFT calculations were carried out using Gaussian 09.[S17]

Computed Cartesian coordinates and energies

***t*-P12** (^{1}A):

```
 BLYP/6-31G(d)  Energy= -35923.436575

C                  12.695507   -2.820423   -5.562617
C                  12.619954   -3.503908   -4.289524
C                  12.745310   -2.543715   -3.312988
C                  12.865190   -1.270188   -3.989612
N                  12.850587   -1.455452   -5.364608
H                  12.500438   -4.577889   -4.165112
H                  12.739365   -2.677958   -2.233706
C                  12.911405   -0.000727   -3.331708
C                  12.865336    1.268737   -3.989617
C                  12.745605    2.542280   -3.312999
N                  12.850753    1.453996   -5.364614
C                  12.620360    3.502484   -4.289539
H                  12.739677    2.676529   -2.233718
C                  12.695833    2.818985   -5.562629
H                  12.500969    4.576479   -4.165131
C                  12.628441    3.464541   -6.837820
C                  12.880982    2.818760   -8.091449
C                  12.990054    3.494593   -9.369190
N                  13.062316    1.459555   -8.249668
C                  13.255965    2.525381  -10.308978
H                  12.887789    4.567833   -9.515742
C                  13.277082    1.255884   -9.610954
H                  13.406801    2.652690  -11.380682
C                  13.407031   -0.000770  -10.221831
C                  13.276939   -1.257407   -9.610948
C                  13.255677   -2.526905  -10.308967
N                  13.062149   -1.461047   -8.249662
C                  12.989654   -3.496081   -9.369174
H                  13.406499   -2.654236  -11.380670
C                  12.880658   -2.820231   -8.091437
H                  12.887266   -4.569310   -9.515722
C                  12.628041   -3.465977   -6.837805
Zn                  12.962418   -0.000737   -6.808174
C                  12.222046   -4.821377   -6.863895
C                  11.693328   -5.944510   -6.873226
C                  12.222603    4.819989   -6.863915
C                  11.694015    5.943182   -6.873249
C                   6.708439   11.552804  -10.217428
C                   5.548056   12.056359   -9.609783
C                   7.734283   10.816554   -9.605207
```

C	4.437936	12.669623	-10.310475
N	5.257039	11.961752	-8.250662
C	8.832024	10.175971	-10.300956
N	7.798239	10.521914	-8.245163
C	3.458566	12.911698	-9.374827
H	4.408179	12.870386	-11.381140
C	3.984641	12.474536	-8.096766
Zn	6.469573	11.144966	-6.807323
C	9.538960	9.461461	-9.361284
H	9.022963	10.250448	-11.371238
C	8.890116	9.692528	-8.085732
H	2.475722	13.353807	-9.524237
C	3.290405	12.564968	-6.846886
N	7.671021	10.319996	-5.362315
N	5.138806	11.753195	-5.367978
H	10.422793	8.843779	-9.506533
C	9.319882	9.145773	-6.833258
C	3.876122	12.292757	-5.570055
C	1.912645	12.886678	-6.878351
C	8.783618	9.513878	-5.558816
C	7.508556	10.414781	-3.987628
C	5.298105	11.663651	-3.992550
C	10.298654	8.124010	-6.860380
C	3.235100	12.552139	-4.299036
C	0.675831	12.991847	-6.890270
C	9.332923	9.102285	-4.285038
C	8.553309	9.678194	-3.309388
C	6.422407	11.075599	-3.331945
C	4.125720	12.180005	-3.319474
C	11.014618	7.109961	-6.871990
H	2.240564	12.975677	-4.178186
H	10.207534	8.467839	-4.159750
H	8.660109	9.599443	-2.229899
H	3.998306	12.230958	-2.240541
C	-11.013789	7.111195	-6.871990
C	-11.693319	5.944494	-6.873249
C	-10.297709	8.125163	-6.860381
C	-12.222041	4.821363	-6.863916
C	-9.318823	9.146816	-6.833262
C	-12.628037	3.465963	-6.837822
C	-8.888992	9.693516	-8.085737
C	-8.782523	9.514869	-5.558820
C	-12.880653	2.820214	-8.091453
C	-12.695504	2.820413	-5.562633
C	-9.537857	9.462513	-9.361290
N	-7.797022	10.522779	-8.245169
C	-9.331877	9.103345	-4.285041
N	-7.669837	10.320865	-5.362320
N	-13.062144	1.461029	-8.249675
C	-12.989648	3.496061	-9.369192
N	-12.850583	1.455442	-5.364620
C	-12.619952	3.503901	-4.289541
C	-8.830837	10.176938	-10.300964
H	-10.421758	8.844927	-9.506538
C	-7.733028	10.817402	-9.605216
Zn	-6.468292	11.145692	-6.807328
C	-8.552203	9.679173	-3.309392
H	-10.206559	8.468997	-4.159752
C	-7.507365	10.415640	-3.987632
C	-13.276932	1.257385	-9.610960
Zn	-12.962413	0.000723	-6.808182
C	-13.255670	2.526881	-10.308982
H	-12.887259	4.569289	-9.515743
C	-12.865187	1.270181	-3.989623
C	-12.745309	2.543710	-3.313003
H	-12.500436	4.577882	-4.165132
H	-9.021764	10.251429	-11.371247
C	-6.707100	11.553534	-10.217438

N	-5.255661	11.962333	-8.250669
N	-5.137462	11.753781	-5.367983
H	-8.659015	9.600440	-2.229903
C	-6.421145	11.076339	-3.331950
C	-13.407023	0.000747	-10.221840
N	-13.062310	-1.459573	-8.249673
N	-12.850749	-1.454007	-5.364619
H	-13.406490	2.654210	-11.380686
C	-12.911403	0.000722	-3.331717
H	-12.739365	2.677956	-2.233722
C	-5.546663	12.056964	-9.609792
C	-3.983207	12.474976	-8.096772
C	-5.296775	11.664263	-3.992555
C	-3.874717	12.293202	-5.570059
C	-13.277075	-1.255905	-9.610959
C	-12.880976	-2.818778	-8.091450
C	-12.695829	-2.818996	-5.562630
C	-12.865333	-1.268744	-3.989622
C	-4.436472	12.670099	-10.310484
C	-3.457078	12.912070	-9.374834
C	-3.288965	12.565338	-6.846890
C	-4.124335	12.180491	-3.319478
C	-3.233671	12.552520	-4.299040
C	-13.255957	-2.525404	-10.308980
C	-12.990047	-3.494613	-9.369189
C	-12.628436	-3.464555	-6.837819
C	-12.620357	-3.502491	-4.289538
C	-12.745603	-2.542285	-3.313001
H	-4.406689	12.870852	-11.381150
H	-2.474184	13.354069	-9.524243
C	-1.911169	12.886894	-6.878352
H	-3.996919	12.231437	-2.240545
H	-2.239088	12.975949	-4.178188
H	-13.406792	-2.652716	-11.380684
H	-12.887782	-4.567854	-9.515738
C	-12.222600	-4.820003	-6.863910
H	-12.500966	-4.576486	-4.165128
H	-12.739674	-2.676531	-2.233719
C	-0.674343	12.991924	-6.890270
C	-11.694014	-5.943197	-6.873242
C	-11.014616	-7.109976	-6.871980
C	-10.298647	-8.124022	-6.860367
C	-9.319875	-9.145783	-6.833243
C	-8.783612	-9.513886	-5.558800
C	-8.890108	-9.692541	-8.085716
C	-9.332917	-9.102291	-4.285023
N	-7.671015	-10.320005	-5.362297
N	-7.798232	-10.521928	-8.245144
C	-9.538951	-9.461476	-9.361268
C	-8.553304	-9.678199	-3.309371
H	-10.207527	-8.467844	-4.159737
C	-7.508551	-10.414788	-3.987609
Zn	-6.469567	-11.144979	-6.807302
C	-7.734275	-10.816569	-9.605188
C	-8.832014	-10.175988	-10.300939
H	-10.422784	-8.843793	-9.506518
H	-8.660105	-9.599446	-2.229883
C	-6.422404	-11.075605	-3.331925
N	-5.138802	-11.753206	-5.367956
N	-5.257033	-11.961768	-8.250640
C	-6.708431	-11.552822	-10.217407
H	-9.022953	-10.250466	-11.371220
C	-5.298102	-11.663659	-3.992527
C	-3.876118	-12.292770	-5.570031
C	-3.984636	-12.474553	-8.096741
C	-5.548049	-12.056378	-9.609760
C	-4.125717	-12.180013	-3.319450
C	-3.235097	-12.552150	-4.299011

C	-3.290400	-12.564983	-6.846861
C	-3.458560	-12.911718	-9.374801
C	-4.437930	-12.669644	-10.310451
H	-3.998304	-12.230965	-2.240517
H	-2.240561	-12.975688	-4.178159
C	-1.912639	-12.886693	-6.878324
H	-2.475717	-13.353828	-9.524210
H	-4.408172	-12.870409	-11.381115
C	-0.675825	-12.991857	-6.890242
C	0.674349	-12.991930	-6.890242
C	1.911174	-12.886895	-6.878323
C	3.288970	-12.565340	-6.846860
C	3.874721	-12.293202	-5.570030
C	3.983213	-12.474981	-8.096742
C	3.233673	-12.552516	-4.299010
N	5.137468	-11.753785	-5.367953
N	5.255668	-11.962342	-8.250639
C	3.457084	-12.912077	-9.374803
C	4.124337	-12.180487	-3.319449
H	2.239089	-12.975940	-4.178160
C	5.296780	-11.664265	-3.992525
Zn	6.468301	-11.145703	-6.807299
C	5.546671	-12.056977	-9.609761
C	4.436479	-12.670110	-10.310453
H	2.474189	-13.354073	-9.524213
H	3.996920	-12.231431	-2.240516
C	6.421151	-11.076344	-3.331921
N	7.669848	-10.320878	-5.362291
N	7.797035	-10.522798	-8.245140
C	6.707111	-11.553553	-10.217407
H	4.406697	-12.870865	-11.381118
C	7.507375	-10.415650	-3.987603
C	8.782537	-9.514886	-5.558791
C	8.889008	-9.693537	-8.085708
C	7.733042	-10.817424	-9.605186
C	8.552214	-9.679185	-3.309362
C	9.331892	-9.103361	-4.285012
C	9.318839	-9.146836	-6.833233
C	9.537874	-9.462539	-9.361261
C	8.830853	-10.176964	-10.300935
H	8.659026	-9.600451	-2.229874
H	10.206576	-8.469016	-4.159724
C	10.297726	-8.125184	-6.860354
H	10.421777	-8.844956	-9.506509
H	9.021781	-10.251457	-11.371217
C	11.013801	-7.111212	-6.871965
C	12.880679	-2.820217	8.091438
C	12.989678	-3.496065	9.369176
C	13.255705	-2.526887	10.308966
C	13.276967	-1.257390	9.610945
N	13.062173	-1.461033	8.249659
H	12.887289	-4.569293	9.515726
H	13.406529	-2.654216	11.380669
C	13.407061	-0.000752	10.221824
C	13.277111	1.255900	9.610945
C	13.255996	2.525399	10.308967
N	13.062342	1.459569	8.249660
C	12.990083	3.494609	9.369178
H	13.406835	2.652710	11.380670
C	12.881006	2.818774	8.091439
H	12.887818	4.567849	9.515728
C	12.628462	3.464552	6.837809
C	12.695851	2.818994	5.562619
C	12.620375	3.502490	4.289528
N	12.850772	1.454005	5.364606
C	12.745617	2.542285	3.312989
H	12.500983	4.576485	4.165119
C	12.865350	1.268743	3.989610

H	12.739686	2.676531	2.233708
C	12.911417	-0.000722	3.331704
C	12.865203	-1.270182	3.989609
C	12.745320	-2.543710	3.312989
N	12.850603	-1.455443	5.364606
C	12.619965	-3.503901	4.289527
H	12.739372	-2.677956	2.233707
C	12.695522	-2.820414	5.562618
H	12.500447	-4.577882	4.165117
C	12.628058	-3.465965	6.837808
Zn	12.962439	-0.000726	6.808168
C	12.222059	-4.821364	6.863902
C	11.693338	-5.944495	6.873238
C	12.222622	4.819999	6.863903
C	11.694030	5.943191	6.873236
C	6.422405	11.075590	3.331932
C	5.298101	11.663639	3.992537
C	7.508555	10.414775	3.987614
C	4.125714	12.179990	3.319461
N	5.138801	11.753182	5.367965
C	8.553311	9.678192	3.309374
N	7.671020	10.319990	5.362302
C	3.235094	12.552122	4.299023
H	3.998301	12.230944	2.240528
C	3.876116	12.292741	5.570042
Zn	6.469570	11.144956	6.807309
C	9.332926	9.102286	4.285025
H	8.660111	9.599443	2.229886
C	8.783620	9.513875	5.558803
H	2.240557	12.975658	4.178172
C	3.290398	12.564951	6.846873
N	7.798237	10.521908	8.245150
N	5.257033	11.961738	8.250649
H	10.207540	8.467843	4.159737
C	9.319885	9.145772	6.833245
C	3.984634	12.474520	8.096753
C	1.912638	12.886660	6.878336
C	8.890117	9.692525	8.085719
C	7.734280	10.816546	9.605194
C	5.548049	12.056346	9.609770
C	10.298661	8.124014	6.860367
C	3.458557	12.911680	9.374814
C	0.675824	12.991830	6.890255
C	9.538961	9.461460	9.361270
C	8.832022	10.175967	10.300943
C	6.708434	11.552793	10.217415
C	4.437928	12.669607	10.310462
C	11.014630	7.109968	6.871977
H	2.475713	13.353787	9.524224
H	10.422796	8.843780	9.506519
H	9.022961	10.250444	11.371225
H	4.408170	12.870370	11.381127
C	-11.013815	7.111209	6.871960
C	-11.693348	5.944509	6.873218
C	-10.297731	8.125175	6.860352
C	-12.222071	4.821380	6.863884
C	-9.318842	9.146824	6.833234
C	-12.628068	3.465980	6.837793
C	-8.782539	9.514876	5.558794
C	-8.889011	9.693522	8.085711
C	-12.695530	2.820426	5.562604
C	-12.880689	2.820233	8.091424
C	-9.331893	9.103354	4.285014
N	-7.669850	10.320868	5.362295
C	-9.537878	9.462521	9.361262
N	-7.797039	10.522782	8.245145
N	-12.850609	1.455455	5.364594
C	-12.619973	3.503911	4.289512

N	-13.062180	1.461049	8.249648
C	-12.989689	3.496084	9.369161
C	-8.552215	9.679179	3.309365
H	-10.206577	8.469008	4.159723
C	-7.507376	10.415643	3.987607
Zn	-6.468304	11.145691	6.807305
C	-8.830858	10.176944	10.300938
H	-10.421782	8.844938	9.506509
C	-7.733046	10.817405	9.605191
C	-12.865207	1.270191	3.989598
Zn	-12.962443	0.000740	6.808160
C	-12.745326	2.543718	3.312975
H	-12.500456	4.577892	4.165100
C	-13.276974	1.257409	9.610933
C	-13.255715	2.526907	10.308952
H	-12.887301	4.569313	9.515709
H	-8.659026	9.600447	2.229877
C	-6.421152	11.076339	3.331927
N	-5.137471	11.753777	5.367962
N	-5.255672	11.962327	8.250648
H	-9.021786	10.251435	11.371220
C	-6.707116	11.553533	10.217414
C	-12.911420	0.000730	3.331695
N	-12.850773	-1.453994	5.364600
N	-13.062345	-1.459552	8.249654
H	-12.739377	2.677962	2.233693
C	-13.407067	0.000772	10.221815
H	-13.406539	2.654238	11.380655
C	-5.296782	11.664259	3.992533
C	-3.874724	12.293193	5.570040
C	-5.546676	12.056959	9.609770
C	-3.983217	12.474967	8.096752
C	-12.865352	-1.268734	3.989604
C	-12.695852	-2.818982	5.562616
C	-12.881009	-2.818758	8.091435
C	-13.277115	-1.255882	9.610938
C	-4.124339	12.180483	3.319459
C	-3.233675	12.552510	4.299021
C	-3.288973	12.565328	6.846872
C	-4.436484	12.670090	10.310464
C	-3.457088	12.912059	9.374815
C	-12.745618	-2.542277	3.312986
C	-12.620375	-3.502481	4.289526
C	-12.628463	-3.464538	6.837806
C	-12.990084	-3.494590	9.369175
C	-13.255999	-2.525379	10.308963
H	-3.996922	12.231429	2.240526
H	-2.239091	12.975935	4.178172
C	-1.911176	12.886880	6.878335
H	-4.406702	12.870843	11.381130
H	-2.474193	13.354055	9.524227
H	-12.739685	-2.676525	2.233704
H	-12.500982	-4.576475	4.165119
C	-12.222625	-4.819985	6.863903
H	-12.887819	-4.567830	9.515728
H	-13.406837	-2.652688	11.380666
C	-0.674350	12.991907	6.890255
C	-11.694035	-5.943178	6.873239
C	-11.014635	-7.109955	6.871983
C	-10.298664	-8.124000	6.860376
C	-9.319889	-9.145759	6.833258
C	-8.890123	-9.692510	8.085733
C	-8.783623	-9.513866	5.558817
C	-9.538969	-9.461440	9.361283
N	-7.798246	-10.521894	8.245167
N	-7.671025	-10.319984	5.362319
C	-9.332927	-9.102278	4.285037
C	-8.832032	-10.175946	10.300959

H	-10.422803	-8.843758	9.506530
C	-7.734290	-10.816529	9.605213
Zn	-6.469578	-11.144949	6.807330
C	-7.508558	-10.414772	3.987632
C	-8.553312	-9.678188	3.309389
H	-10.207539	-8.467833	4.159747
H	-9.022972	-10.250420	11.371240
C	-6.708446	-11.552776	10.217436
N	-5.257044	-11.961729	8.250673
N	-5.138809	-11.753181	5.367988
C	-6.422409	-11.075591	3.331952
H	-8.660111	-9.599441	2.229901
C	-5.548062	-12.056333	9.609794
C	-3.984646	-12.474513	8.096779
C	-3.876125	-12.292742	5.570068
C	-5.298107	-11.663641	3.992559
C	-4.437942	-12.669593	10.310488
C	-3.458571	-12.911670	9.374842
C	-3.290408	-12.564948	6.846900
C	-3.235102	-12.552127	4.299050
C	-4.125721	-12.179996	3.319486
H	-4.408185	-12.870353	11.381154
H	-2.475727	-13.353778	9.524254
C	-1.912647	-12.886656	6.878366
H	-2.240566	-12.975665	4.178201
H	-3.998307	-12.230953	2.240553
C	-0.675833	-12.991820	6.890286
C	0.674341	-12.991893	6.890287
C	1.911166	-12.886859	6.878367
C	3.288963	-12.565305	6.846904
C	3.983206	-12.474942	8.096784
C	3.874715	-12.293175	5.570072
C	3.457076	-12.912029	9.374848
N	5.255662	-11.962304	8.250680
N	5.137462	-11.753761	5.367993
C	3.233666	-12.552494	4.299054
C	4.436471	-12.670060	10.310497
H	2.474180	-13.354023	9.524260
C	5.546665	-12.056932	9.609802
Zn	6.468297	-11.145675	6.807335
C	5.296775	-11.664248	3.992564
C	4.124331	-12.180472	3.319490
H	2.239081	-12.975918	4.178205
H	4.406688	-12.870809	11.381164
C	6.707105	-11.553507	10.217445
N	7.797032	-10.522765	8.245173
N	7.669845	-10.320859	5.362323
C	6.421146	-11.076332	3.331957
H	3.996914	-12.231421	2.240557
C	7.733038	-10.817384	9.605220
C	8.889007	-9.693508	8.085737
C	8.782535	-9.514868	5.558820
C	7.507371	-10.415636	3.987635
C	8.830851	-10.176923	10.300966
C	9.537874	-9.462505	9.361288
C	9.318839	-9.146814	6.833259
C	9.331891	-9.103351	4.285039
C	8.552212	-9.679177	3.309392
H	9.021778	-10.251412	11.371249
H	10.421779	-8.844924	9.506534
C	10.297729	-8.125165	6.860375
H	10.206576	-8.469007	4.159747
H	8.659023	-9.600448	2.229903
C	11.013808	-7.111196	6.871981
C	-6.428552	-11.091417	1.915294
C	-6.433019	-11.101022	0.675762
C	-6.428548	-11.091425	-1.915267
C	-6.433018	-11.101024	-0.675735

C	-12.936599	0.000724	-1.915237
C	-12.949965	0.000726	-0.675744
C	-12.936609	0.000728	1.915215
C	-12.949969	0.000728	0.675721
C	-6.427289	11.092166	-1.915292
C	-6.431759	11.101769	-0.675760
C	-6.427294	11.092166	1.915269
C	-6.431760	11.101769	0.675737
C	6.428551	11.091420	-1.915287
C	6.433019	11.101019	-0.675755
C	6.428549	11.091413	1.915274
C	6.433018	11.101017	0.675742
C	12.936600	-0.000725	-1.915228
C	12.949965	-0.000725	-0.675735
C	12.936607	-0.000723	1.915223
C	12.949968	-0.000724	0.675730
C	6.427294	-11.092169	-1.915263
C	6.431760	-11.101770	-0.675730
C	6.427289	-11.092162	1.915299
C	6.431758	-11.101767	0.675766
H	-6.790612	11.696701	11.298617
H	6.791955	11.695971	-11.298629
H	-6.790595	11.696703	-11.298640
H	-13.564570	0.000756	-11.304245
H	-6.791946	-11.695991	-11.298608
H	6.790607	-11.696724	-11.298609
H	13.564579	-0.000781	-11.304236
H	6.791948	11.695960	11.298617
H	13.564612	-0.000762	11.304229
H	6.790601	-11.696673	11.298648
H	-6.791962	-11.695940	11.298638
H	-13.564618	0.000783	11.304220

Herzberg-Teller vibration

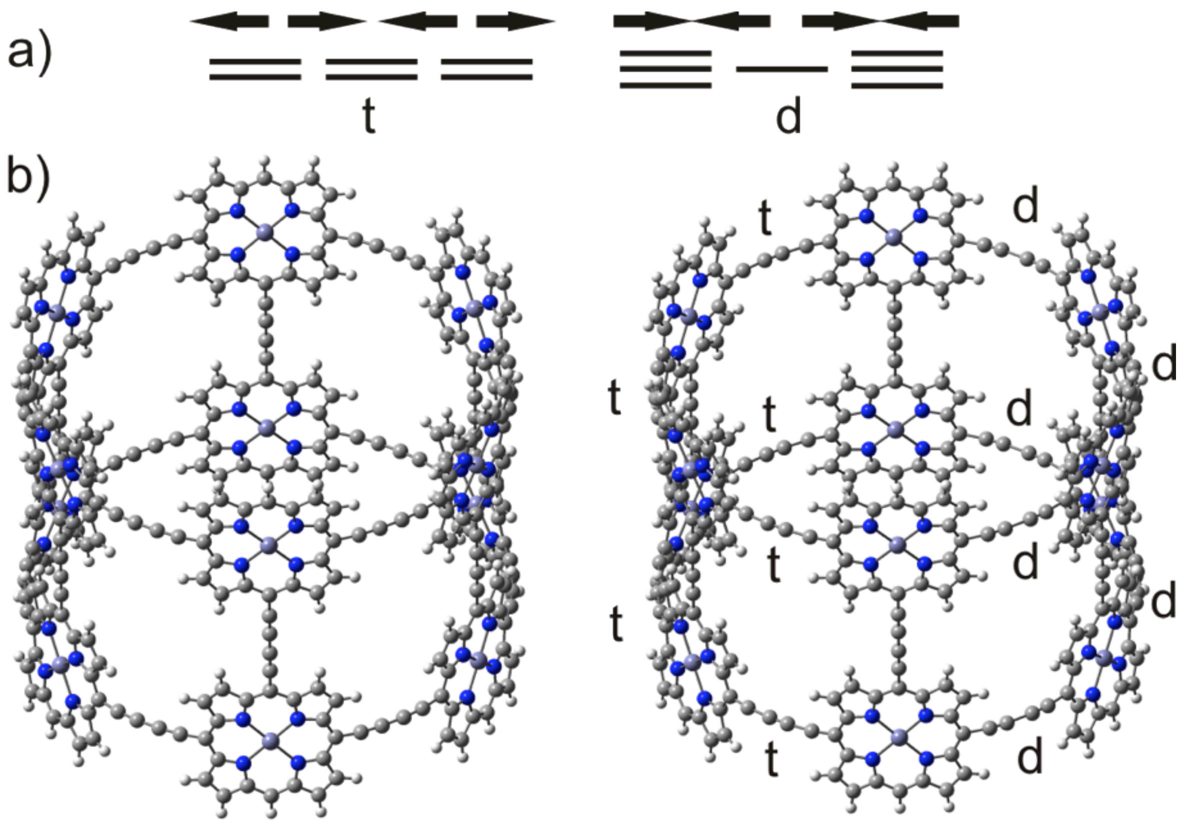

Figure S3. a) Direction of the change in bond length alternation during the butadiyne stretching vibration of ***t*-P12**, giving rise to a more hexatriene-like structure (labeled **t**, left side) or a butadiyne structure (labeled **d**, right side). b) Left side: Ground state structure of ***t*-P12**. Right side: Distortion (labels according to the description in a)) of the butadiyne bonds during the HT active vibration of **1**. The stretching vibration is calculated to be found at 2160 cm^{-1} at the BLYP/6-31G(d) level of theory.

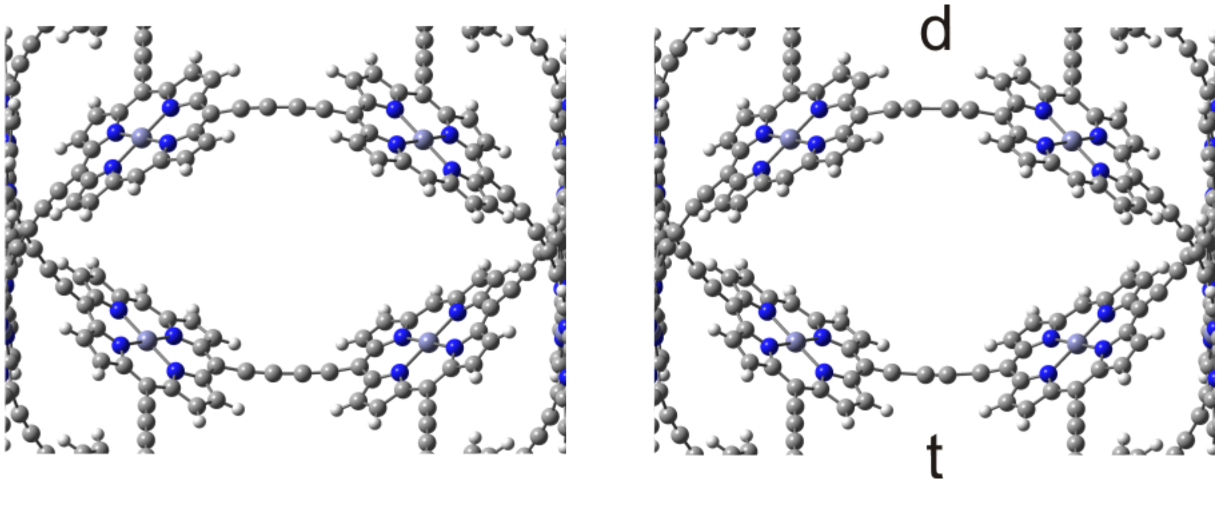

Figure S4. Left side: Ground state structure of ***t*-P12**. Right side: Zoom-in of the distortion (labels according to the description in Figure S3, only the distortions of two oppositely situated butadiynes of ***t*-P12** are shown for clarity, image is the same as on the left side) of the butadiyne bonds during a HT active vibration of ***t*-P12**. This stretching vibration is calculated to be found at 2160 cm^{-1} at the BLYP/6-31G(d) level of theory.

Photoluminescence Upconversion Spectroscopy

The photoluminescence (PL) upconversion technique was used to investigate fluorescence dynamics of sample solutions held in quartz cuvettes as described in detail elsewhere.[S18-S19]

An excitation pulse was generated by a mode-locked Ti:Sapphire laser with pulse duration of 100 fs and a repetition rate of 80 MHz. Fluorescence is collected and optically gated in a beta-barium-oxide (BBO) crystal by a vertically polarized time-delayed gate beam. The upconverted signal, which consists of sum-frequency photons from the gate pulse and the vertical component of the fluorescence, was collected, dispersed in a monochromator and detected using a nitrogen-cooled CCD. Using a combination of a half-wave plate and a Glan-Thompson polarizer, the polarization of the excitation pulse was varied and fluorescence intensity dynamics were recorded separately for components polarized parallel $I_{\parallel}$ and perpendicular $I_{\perp}$ to the excitation pulse polarization. The fluorescence anisotropy is defined using $\gamma = (I_{\parallel} - I_{\perp})/(I_{\parallel} + 2I_{\perp})$ and calculated from the measured components. The full-width-half-maximum of the instrumental response function was measured to be 270 fs. No anisotropy decay was observed, indicating that any polarization decay must occur within 200 fs for this level of signal-to-noise.

The concentration of the sample solution was on the order of 10^{-4} mol/L. The excitation fluence was kept low (0.32 μJ/cm^2) to avoid fluorescence quenching via exciton-exciton annihilation. The detection wavelength was 941 nm.

References

[S1] F. C. Grozema, C. Houarner-Rassin, P. Prins, L. D. A. Siebeles, H. L. Anderson, *J. Am. Chem. Soc.* **2007**, *129*, 13370–13371.

[S2] M. Hoffmann, J. Kärnbratt, M.-H. Chang, L. M. Herz, B. Albinsson, H. L. Anderson, *Angew. Chem. Int. Ed.* **2008**, *47*, 4993–4996.

[S3] T. E. O. Screen, K. B. Lawton, G. S. Wilson, N. Dolney, R. Ispasoiu, T. Goodson III, S. J. Martin, D. D. C. Bradley, H. L. Anderson, *J. Mater. Chem.* **2001**, *11*, 312–320.

[S4] H. M. Marques, I. Cukrowski, *Phys. Chem. Chem. Phys.* **2003**, *5*, 5499–5506.

[S5] H. M. Marques, I. Cukrowski, *Phys. Chem. Chem. Phys.* **2002**, *4*, 5878–5887.

[S6] E. Goldstein, B. Ma, J.-H. Lii, N. L. Allinger, *J. Phys. Org. Chem.* **1996**, *9*, 191–202.

[S7] P. D. Jarowski, F. Diederich, K. N. Houk, *J. Org. Chem.* **2005**, *70*, 1671–1678.

[S8] A. D. Becke, *Phys. Rev. A* **1988**, *38*, 3098.

[S9] C. Lee, W. Yang, R. G. Parr, *Physical Review B: Condensed Matter and Materials Physics* **1988**, *37*, 785.

[S10] B. Miehlich, A. Savin, H. Stoll, H. Preuss, *Chem. Phys. Lett.* **1989**, *157*, 200.

[S11] R. Ditchfield, W. J. Hehre, J. A. Pople, *J. Chem. Phys.* **1971**, *54*, 724.

[S12] W. J. Hehre, R. Ditchfield, J. A. Pople, *J. Chem. Phys.* **1972**, *56*, 2257.

[S13] M. M. Francl, W. J. Pietro, W. J. Hehre, J. S. Binkley, D. J. DeFrees, J. A. Pople, M. S. Gordon, *J. Chem. Phys.* **1982**, *77*, 3654.

[S14] R. C. Binning Jr., L. A. Curtiss, *J. Comp. Chem.* **1990**, *11*, 1206.

[S15] V. A. Rassolov, J. A. Pople, M. A. Ratner, T. L. Windus, *J. Chem. Phys.* **1998**, *109*, 1223.

[S16] V. A. Rassolow, M. A. Ratner, J. A. Pople, P. C. Redfern, L. A. Curtiss, *J. Comp. Chem.* **2001**, *22*, 976.

[S17] G. W. T. M. J. Frisch, H. B. Schlegel, G. E. Scuseria, J. R. C. M. A. Robb, J. A. Montgomery, Jr., T. Vreven, J. C. B. K. N. Kudin, J. M. Millam, S. S. Iyengar, J. Tomasi, , B. M. V. Barone, M. Cossi, G. Scalmani, N. Rega, H. N. G. A. Petersson, M. Hada, M. Ehara, K. Toyota, J. H. R. Fukuda, M. Ishida, T. Nakajima, Y. Honda, O. Kitao, M. K. H. Nakai, X. Li, J. E. Knox, H. P. Hratchian, J. B. Cross, J. J. C. Adamo, R. Gomperts, R. E. Stratmann, O. Yazyev, R. C. A. J. Austin, C. Pomelli, J. W. Ochterski, P. Y. Ayala, G. A. V. K. Morokuma, P. Salvador, J. J. Dannenberg, S. D. V. G. Zakrzewski, A. D. Daniels, M. C. Strain, D. K. M. O. Farkas, A. D. Rabuck, K. Raghavachari, J. V. O. J. B. Foresman, Q. Cui, A. G. Baboul, S. Clifford, B. B. S. J. Cioslowski, G. Liu, A. Liashenko, P. Piskorz, R. L. M. I. Komaromi, D. J. Fox, T. Keith, M. A. Al-Laham, A. N. C. Y. Peng, M. Challacombe, P. M. W. Gill, W. C. B. Johnson, M. W. Wong, C. Gonzalez, J. A. Pople, Gaussian, Inc., Pittsburgh PA, **2003**.

[S18] M. H. Chang, F. J. M. Hoeben, P. Jonkheijm, A. P. H. J. Schenning, E. W. Meijer, C. Silva, L. M. Herz, *Chem. Phys. Lett.* **2006**, *418*, 196–201.

[S19] M. H. Chang, M. Hoffmann, H. L. Anderson, L. M. Herz, *J. Am. Chem. Soc.* **2008**, *130*, 10171–10178.
